# In vivo O_2_ imaging in hepatic tissues by phosphorescence lifetime imaging microscopy using Ir(III) complexes as intracellular probes

**DOI:** 10.1038/s41598-020-76878-6

**Published:** 2020-12-03

**Authors:** Kiichi Mizukami, Ayaka Katano, Shuichi Shiozaki, Toshitada Yoshihara, Nobuhito Goda, Seiji Tobita

**Affiliations:** 1 Department of Chemistry and Chemical Biology, School of Science and Technology, Gunma University, Kiryu, Gunma 376-8515 Japan; 2 Department of Life Science and Medical BioScience, School of Advanced Science and Engineering, Waseda University, Wakamatsu-cho, Shinjuku-ku, Tokyo, 162-8480 Japan

**Keywords:** Biological techniques, Chemical biology, Chemistry

## Abstract

Phosphorescence lifetime imaging microscopy (PLIM) combined with an oxygen (O_2_)-sensitive luminescent probe allows for high-resolution O_2_ imaging of living tissues. Herein, we present phosphorescent Ir(III) complexes, (btp)_2_Ir(acac-DM) (**Ir-1**) and (btp-OH)_3_Ir (**Ir-2**), as useful O_2_ probes for PLIM measurement. These small-molecule probes were efficiently taken up into cultured cells and accumulated in specific organelles. Their excellent cell-permeable properties allowed for efficient staining of three-dimensional cell spheroids, and thereby phosphorescence lifetime measurements enabled the evaluation of the O_2_ level and distribution in spheroids, including the detection of alterations in O_2_ levels by metabolic stimulation with an effector. We took PLIM images of hepatic tissues of living mice by intravenously administrating these probes. The PLIM images clearly visualized the O_2_ gradient in hepatic lobules with cellular-level resolution, and the O_2_ levels were derived based on calibration using cultured cells; the phosphorescence lifetime of **Ir-1** gave reasonable O_2_ levels, whereas **Ir-2** exhibited much lower O_2_ levels. Intravenous administration of NH_4_Cl to mice caused the hepatic tissues to experience hypoxia, presumably due to O_2_ consumption to produce ATP required for ammonia detoxification, suggesting that the metabolism of the probe molecule might affect liver O_2_ levels.

## Introduction

Molecular oxygen (O_2_) is essential for cellular function and is continuously supplied to whole tissues in the body to maintain homeostasis. Oxygen deprivation (hypoxia) in tissues is associated with the pathophysiology of various diseases such as cerebral infarction, fatty liver disease, chronic kidney disease, diabetic retinopathy, and cancer^1–3^. Understanding the cellular and molecular mechanisms underlying hypoxia-associated diseases requires O_2_ imaging technology capable of detecting the tissue oxygen status in real time and with high resolution. In vivo O_2_ detection methods that have been developed so far include oxygen electrodes^4,5^, blood oxygenation level dependent magnetic resonance imaging (Bold MRI)^6,7^, positron emission tomography (PET) with a hypoxia tracer^8^, electron paramagnetic resonance (EPR) oximetry^9,10^, hypoxia markers^11–13^, and optical imaging^14–16^. These methods can work in vivo, but they have advantages and limitations in terms of applicable targets, spatial resolution, tissue permeability, convenience, reversibility, etc. Of these known methods, optical imaging utilizing O_2_ quenching of probe phosphorescence has great advantages in that high-resolution O_2_ images can be obtained in a reversible manner, although it is limited to the detection of a penetration depth through tissues of ~ 1 cm even under near-infrared excitation^17^.

To visualize tissue O_2_ levels with high sensitivity and high stability, various phosphorescent metal complexes have been developed over the past few decades^14–16,18,19^. The most common compounds used as luminescent O_2_ probes include Pt(II) and Pd(II) porphyrins, Ru(II) complexes, Pt(II) complexes, and Ir(III) complexes, which give intense phosphorescence in the visible to near-infrared wavelength regions with reasonably long lifetimes (> 1.0 μs). Oxygen probes targeting various biological tissues of interest have been developed by modifying these O_2_-sensing luminophores: cell-penetrating conjugates of Pt(II)-porphyrins^20,21^, dendritic Pt(II)- and Pd(II)-porphyrins^22–24^, Ru(II) complex derivatives targeting the cell nucleus^25^, ratiometric O_2_ probes based on Ir(III) complexes^26,27^, etc.

With the use of phosphorescent probes, wide-field luminescence lifetime measurements using a gated CCD camera have enabled whole-body O_2_ imaging including tumor tissues, ocular fundus, and specific organs^28–31^. In contrast, phosphorescence lifetime imaging microscopy (PLIM)^32,33^ allows high-resolution O_2_ imaging at the cellular level, providing precise information on microenvironment along with real-time changes in O_2_ levels. The PLIM method has been applied to O_2_ imaging of giant cells^34^, cell spheroids^20,24,35,36^, neurospheres^37,38^ and the epithelium of rat and human colon tissues^38^ using cell-permeable small-molecule and nano-particle probes. By using dendrimerized Pt-porphyrin probes, the PLIM method has been successfully applied to microvascular and interstitial O_2_ imaging of the brain^22,39,40^, bone marrow^41,42^, and retinal tissue^43^ in vivo.

**Figure 1:**
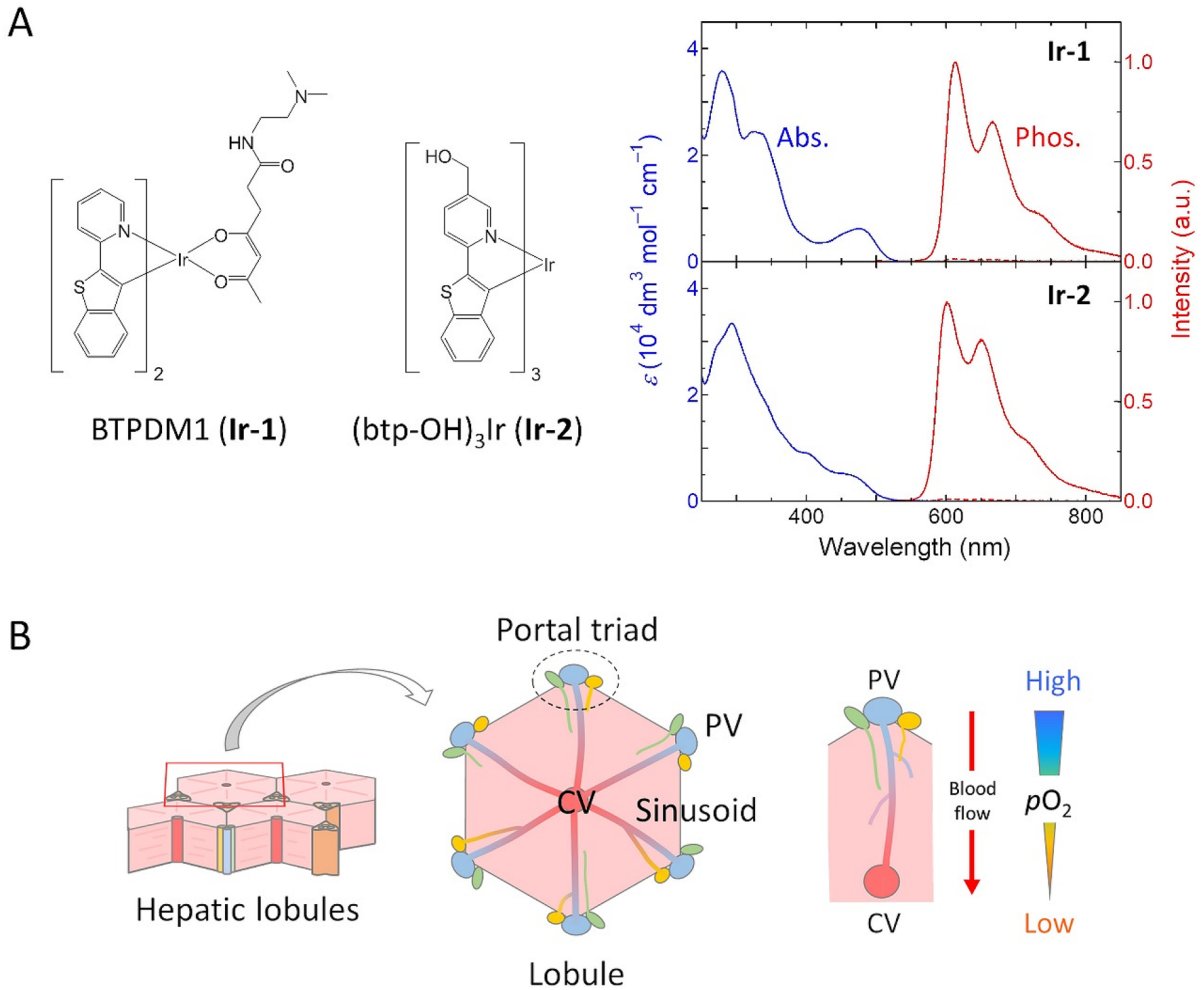
(**A**) Chemical structures of BTPDM1 (**Ir-1**) and (btp-OH)_3_Ir (**Ir-2**). Absorption and phosphorescence spectra of **Ir-1** and **Ir-2** in MeCN at room temperature are shown. The phosphorescence spectra were taken in both degassed (solid line) and aerated (dashed line) solutions. (**B**) Schematic views of hepatic lobules. CV central vein, PV portal vein.

In contrast, few studies have reported tissue oxygen distributions in vivo using an intracellular O_2_ probe^44^. We have recently developed small-molecule O_2_ probes based on Ir(III) complexes^18,19^. Among them, BTPDM1 ((btp)_2_Ir(acac-DM); **Ir-1**) (Fig. 1A) allows for the measurement of O_2_ levels in hypoxic tumors^45,46^ and high-resolution O_2_ imaging in renal cortex in vivo^47,48^. To improve the reliability of the O_2_ level as measured with a small-molecule probe, it is necessary to quantify O_2_ levels in different tissues in vivo and investigate various issues: localization and stability of probes in tissues, calibration of emission lifetime in tissues (cell-specific calibration), the influence of probe administration on the target tissues, etc.

In this study, we established an in vivo O_2_ imaging method using small-molecule probes by investigating the O_2_ distribution in the hepatic lobules of living mice by PLIM measurements with two structurally related Ir(III) complexes, **Ir-1** and (btp-OH)_3_Ir (**Ir-2**) (Fig. 1A). The hepatic lobule is a fundamental structural unit of the liver with a hexagonal shape (Fig. 1B)^49,50^. In the lobule, blood runs unidirectionally from the vertices of the hexagon called portal triads, through microcirculatory networks known as sinusoids, to a central vein (CV) in the middle of the lobule. The portal triad contains two distinct inlets for blood flow: a well-oxygenated hepatic artery and a poorly-oxygenated portal vein, the latter of which constitutes ~ 70% of total liver blood flow. Therefore, even under physiological conditions, liver parenchymal cells and hepatocytes surrounding the portal triad are exposed to relatively low oxygen concentrations, and pericentral hepatocytes experience much lower oxygen concentrations, forming a zonal heterogenous distribution of oxygen along the sinusoids in the hepatic lobule. These intralobular oxygen distributions, however, are considered to be prerequisite for normal liver metabolic functions in a zone-specific manner. In addition, this metabolic zonation is often disrupted in disease conditions with aberrant oxygen distribution in the hepatic lobules. To deepen our knowledge about the dynamic alterations in liver metabolism in both physiological and pathophysiological conditions, real-time imaging of intracellular oxygen tension in situ is a promising method with the development of oxygen-sensitive probes.

We first compared the photophysical properties and oxygen sensitivity of **Ir-1** and **Ir-2** in solution and cultured cells. Next, we investigated the feasibility of these probes for the measurements of O_2_ distribution in cell spheroids. Based on these results, we performed PLIM measurements of hepatic tissues of mice using **Ir-1** and **Ir-2** to reveal the probe performances.

## Results

### Photophysical properties

Emission brightness and O_2_ sensitivity are basic characteristics required for an optical O_2_ probe. The emission brightness of the probes under the same concentration can be evaluated by the product (*ε*Φ_p_^0^) of the molar absorption coefficient (*ε*) at the excitation wavelength and the phosphorescence quantum yield (Φ_p_^0^) under the absence of oxygen, and the O_2_ sensitivity by *k*_q_τ_p_^0^, where *k*_q_ and τ_p_^0^ are the bimolecular quenching rate constant by O_2_ and the phosphorescence lifetime under the absence of oxygen. The first absorption band of **Ir-1** appears at around 476 nm in MeCN (Fig. 1A), which originates from singlet metal-to-ligand-charge-transfer (^1^MLCT) transition. This band is slightly blue-shifted in **Ir-2**, giving a smaller *ε* value (2800 dm^3^mol^−1^ cm^−1^) compared with that (5400 dm^3^mol^−1^ cm^−1^) of **Ir-1** at the excitation wavelength (488 nm) in our PLIM measurements. **Ir-1** and **Ir-2** exhibit red emission extending to the near infrared region and give vibrational structures with maximum wavelengths at 612 and 601 nm, respectively. Both complexes are highly emissive in MeCN (Φ_p_^0^ = 0.26 for **Ir-1** and 0.28 for **Ir-2**). Judging from the *ε* values at 488 nm and the almost equal Φ_p_^0^, **Ir-1** is superior to **Ir-2** in brightness for excitation at 488 nm.

The O_2_ sensitivity of a probe in solution can be evaluated experimentally from the ratio (τ_p_^0^/τ_p_) of the phosphorescence lifetimes under deaerated and aerated conditions. The obtained τ_p_^0^/τ_p_ values of **Ir-1** and **Ir-2** in MeCN were 72 and 97, and the *k*_q_ values were calculated to be 7.59 × 10^4^ mmHg^−1^ s^−1^ for **Ir-1** and 8.22 × 10^4^ mmHg^−1^ s^−1^ for **Ir-2** (Table 1). Using the solubility of oxygen in MeCN at an oxygen partial pressure (*p*O_2_) of 0.21 atm^51^, the quenching rate constant can be converted from pressure units to concentration units. The converted *k*_q_ values (6.38 × 10^9^ M^−1^ s^−1^ for **Ir-1** and 6.91 × 10^9^ M^−1^ s^−1^ for **Ir-2**) were close to the diffusion-controlled rate of bimolecular reactions in MeCN^51^, indicating that oxygen has very high quenching ability to the excited triplet state of both complexes. We recently revealed that the phosphorescence quenching of Ir(III) complexes by molecular oxygen occurs not only by energy transfer but also by charge transfer from the triplet Ir(III) complex to O_2_^52^. As a result, Ir(III) complexes show much larger *k*_q_ values compared with metalloporphyrins and Ru(II) complexes. The O_2_ sensitivity is slightly higher in **Ir-2** than **Ir-1** owing to the longer intrinsic lifetime and larger *k*_q_ value of **Ir-2**. The intrinsic lifetimes (5–10 μs) of **Ir-1** and **Ir-2** are shorter than those (10–100 μs) of Pt-porphyrins, but longer than those of typical Ru(II) complexes used as O_2_ probes^18^, and thus the O_2_ sensitivity of **Ir-1** and **Ir-2** is considered to be comparable with Pt-porphyrins and higher than Ru(II) complexes.

**Table 1:** Photophysical parameters of **Ir-1** and **Ir-2** in MeCN at room temperature.^a^.

Probe	$\uplambda _{{{\text{abs}}}}^{\max } \,({\text{nm}})$	$\uplambda _{{{\text{phos}}}}^{\max } \,({\text{nm}})$	$\uptau _{{\text{p}}}^{0} \,(\upmu {\text{s}})$	$\uptau _{{\text{p}}} \,({\text{ns}})$	$\uptau _{{\text{p}}}^{0} /\uptau _{{\text{p}}}$	$\upphi _{{\text{p}}}^{0}$	$\upphi _{{\text{p}}}$	*k*_q_ (10^4^ mmHg^−1^ s^−1^)
**Ir-1**	476	612	5.86	81.4	72	0.26	0.006	7.59
**Ir-2**	–	601	7.31	75.4	97	0.28	0.005	8.22

$\tau_{{\text{p}}}^{0}$ and ${\Phi }_{{\text{p}}}^{0}$ denote the phosphorescence lifetime and quantum yield taken in degassed solutions, and $\tau_{{\text{p}}}$ and ${\Phi }_{{\text{p}}}$ denote the phosphorescence lifetime and quantum yield taken in aerated solutions.

### Characterization of intracellular O_2_ probes

To evaluate the cellular uptake efficiencies of **Ir-1** and **Ir-2**, we compared the emission intensities of human colorectal adenocarcinoma (HT-29) cells incubated with these complexes (5 μM, incubation time: 2 h) using a plate reader. Here, we used their prototype complex BTP ((btp)_2_Ir(acac); btp = benzothienylpyridine, acac = acetylacetone)^45^ as a reference compound. The emission intensities of each complex under 21% and 2.5% O_2_ conditions were corrected for the number of HT-29 cells in each well and the *ε* values (4700, 5400, and 2800 M^−1^ cm^−1^ for BTP, **Ir-1** and **Ir-2**) at 488 nm. The relative emission intensities after this correction (Fig. S1) suggest that the cellular uptake efficiency was greatly improved with **Ir-1** and **Ir-2** compared with BTP by introducing a hydrophilic dimethylamino and hydroxyl group, respectively.

The subcellular localization of **Ir-1** and **Ir-2** investigated by costaining experiments with organelle-specific trackers (LysoTracker Green and ERTracker Green) using HeLa cells showed preferential accumulation to lysosomes for **Ir-1** and endoplasmic reticulum (ER) for **Ir-2** (Fig. S2). Both complexes taken up into cells are presumed to accumulate in the organelle membrane because of their lipophilicity. The cytotoxicity of **Ir-1** and **Ir-2** evaluated with WST assay (incubation time: 24 h under the presence of the probe) revealed that the median lethal doses (LC_50_) of **Ir-1** and **Ir-2** for HT-29 cells were 10–15 μM and ~ 30 μM (Fig. S3A). In addition, analysis of mitochondrial membrane potential using JC-1 dye (Fig. S3B) showed that the membrane potential was almost unaffected by **Ir-1** or **Ir-2** loading (1 μM, 2 h). In typical experiments in this study, the cells were incubated with the probes (500 nM or 1 μM) for 2 h prior to microscopic measurements. Therefore, under these experimental conditions, the effect of the probes on cell activity is considered to be sufficiently small.

Photostability is another important characteristic required for an intracellular O_2_ probe. We investigated the photostability of **Ir-1** and **Ir-2** in cells by taking emission images of HT-29 cells under continuous 488 nm laser pulse irradiation with our PLIM system. The cells were incubated with **Ir-1** or **Ir-2** (1 μM) for 2 h under 21% or 2.5% O_2_ conditions, and PLIM images of the HT-29 cells were taken at every 50 scans until 550 scans (Fig. S4). During this irradiation time, the phosphorescence lifetimes of **Ir-1** and **Ir-2** were almost constant, although the phosphorescence intensity changed slightly by 488 nm light irradiation. Also, no significant change was observed in the cell morphology. In this study, the signals accumulated with 50 scans were usually averaged to obtain a single PLIM image. Therefore, **Ir-1** and **Ir-2** were found to have sufficient photostability for obtaining clear PLIM images.

### Quantification of O_2_ levels

**Figure 2:**
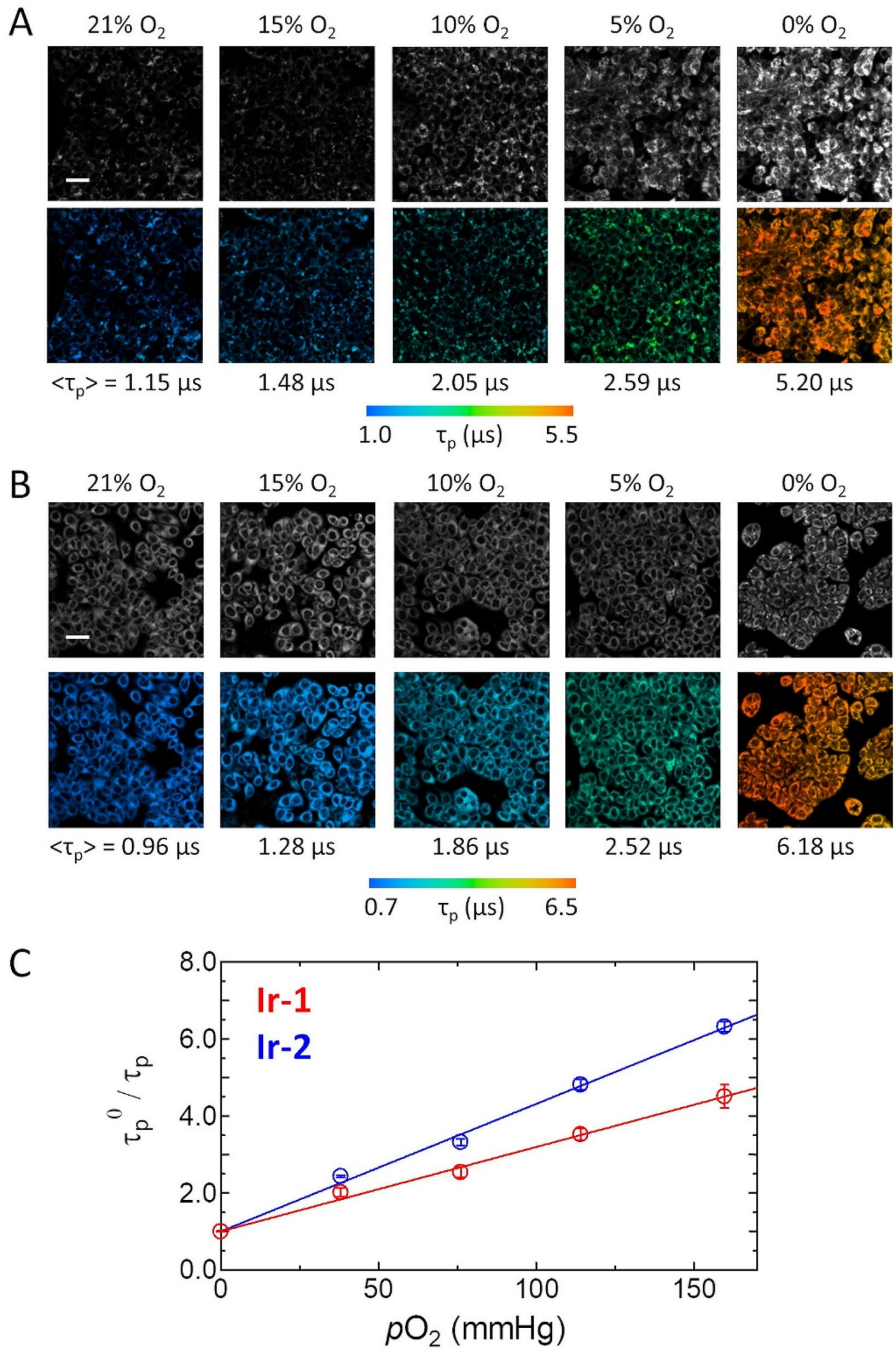
Intensity (upper) and PLIM (lower) images of HT-29 cells stained with (**A**) **Ir-1** and (**B**) **Ir-2** under different *p*O_2_ in an incubator. The average phosphorescence lifetimes are shown below each image. Cells were treated with Ant A (5–21% O_2_) and Na_2_SO_3_ (0% O_2_). Scale bar: 50 µm. (**C**) Stern–Volmer plots of $\tau_{{\text{p}}}^{0}$/$\tau_{{\text{p}}}$ as a function of *p*O_2_ for **Ir-1** (red) and **Ir-2** (blue) partitioned into HT-29 cells under different *p*O_2_ in an incubator. Error bars: S.D.

To quantify the oxygen levels in tissues based on lifetime measurements, we need a calibration curve that represents the relationship between lifetime and *p*O_2_. Since **Ir-1** and **Ir-2** are likely to accumulate in specific organelle membranes, the *p*O_2_ dependence of the phosphorescence lifetime in cells is expected to be different from that in solution. Therefore, we calibrated the lifetime by measuring PLIM images of HT-29 cells incubated with **Ir-1** or **Ir-2** under 21, 15, 10, 5, and 0% O_2_ conditions at 37 °C (Fig. 2A,B); we added 10 µM antimycin A (AntA) to the medium prior to the PLIM measurements to suppress the oxygen consumption by cellular respiration, and in the experiments under N_2_ saturated conditions we added Na_2_SO_3_ (500 mM) into the medium to remove oxygen remaining in the culture. The average lifetime of an entire image was plotted against the *p*O_2_ according to the Stern–Volmer equation, τ_p_^0^/τ_p_ = 1 + *k*_q_ τ_p_^0^*p*O_2_. A linear relationship was obtained for both complexes (Fig. 2C), and the *k*_q_ values for **Ir-1** and **Ir-2** were derived to be 4.22 × 10^3^ mmHg^−1^ s^−1^ and 5.36 × 10^3^ mmHg^−1^ s^−1^ along with the τ_p_^0^, 5.20 µs and 6.18 µs, respectively. These values were used to quantify oxygen levels in cell spheroids from phosphorescence lifetimes (τ_p_).

### Evaluation of O_2_ distribution in cell spheroids

In the past decade, three-dimensional (3D) spheroid systems have received much attention in fields such as drug discovery, cancer research, and toxicology. They provide a more physiologically-relevant environment and organ-like microarchitecture compared with conventional 2D cell cultures and better mimic the crucial tumor tissue properties and microenvironment. The excellent cell-permeable properties of **Ir-1** and **Ir-2** suggested their potential for in-depth staining of 3D cell spheroids and thus the potential of these probes for visualization of the O_2_ distribution within spheroids and living tissues.

**Figure 3:**
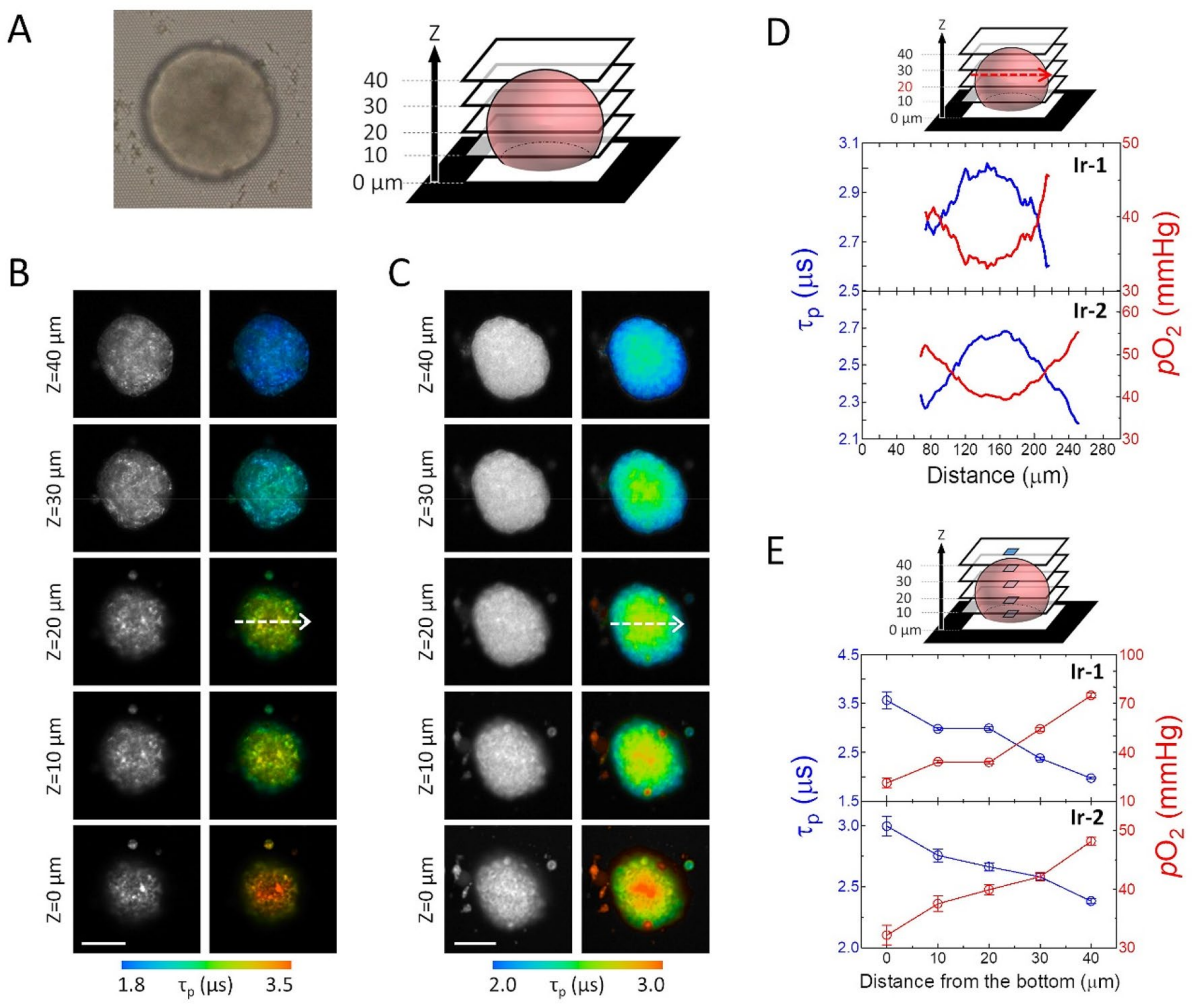
(**A**) Bright-field image (left) and schematic view (right) of an HT-29 cell spheroid. (**B, C**) Z-stacked phosphorescence intensity (left) and lifetime (right) images of an HT-29 cell spheroid stained with (**B**) **Ir-1** and (**C**) **Ir-2**. Each image corresponds to cross-section from the bottom to the upper part at an interval of 10 µm along the z-axis. Scale bar: 100 µm. (**D**) Line profiles of phosphorescence lifetime (blue) and *p*O_2_ (red) along the arrows shown in **B** and **C**. (**E**) Average phosphorescence lifetime (blue) and *p*O_2_ (red) of the square region along the z-axis in an HT-29 cell spheroid stained with probes.

So, we first acquired the PLIM images of HT-29 cell spheroids that were incubated with **Ir-1** or **Ir-2**. Here, each probe (500 nM) was added to the medium after the spheroids were formed, and the spheroids were further incubated with the probe for 24 h prior to PLIM measurements. The bright-field images taken on different planes in the z direction (Fig. 3) show that spheroids with a diameter of 150–200 μm were almost uniformly stained, including the core, by **Ir-1** and **Ir-2**. We found from the Z-stack phosphorescence lifetime images of an HT-29 spheroid stained with **Ir-1** (Fig. 3B) and **Ir-2** (Fig. 3C) that the cells closer to the center of the spheroid gave longer lifetimes, i.e. lower O_2_ levels compared with those in the peripheral region. For quantitative evaluation of the O_2_ gradient in a spheroid, we investigated the line profiles of the average phosphorescence lifetime on the plane approximately 20 μm from the bottom (Fig. 3D). We also measured the lifetimes along the central area in the z-direction (Fig. 3E), and derived the *p*O_2_ distribution (Fig. 3D,E) based on the τ_p_^0^ and *k*_q_ obtained for HT-29 cultured cells. Both probes showed that the *p*O_2_ is reduced near the center and bottom surface of the spheroids, because the oxygen supply from the bottom direction is cut off by the bottom glass, and also the peripheral cells consume oxygen carried by diffusion from the culture medium. The degree of hypoxia in the core depended on the spheroid size (Fig. S5) and the respiratory activity as will be described below.

**Figure 4:**
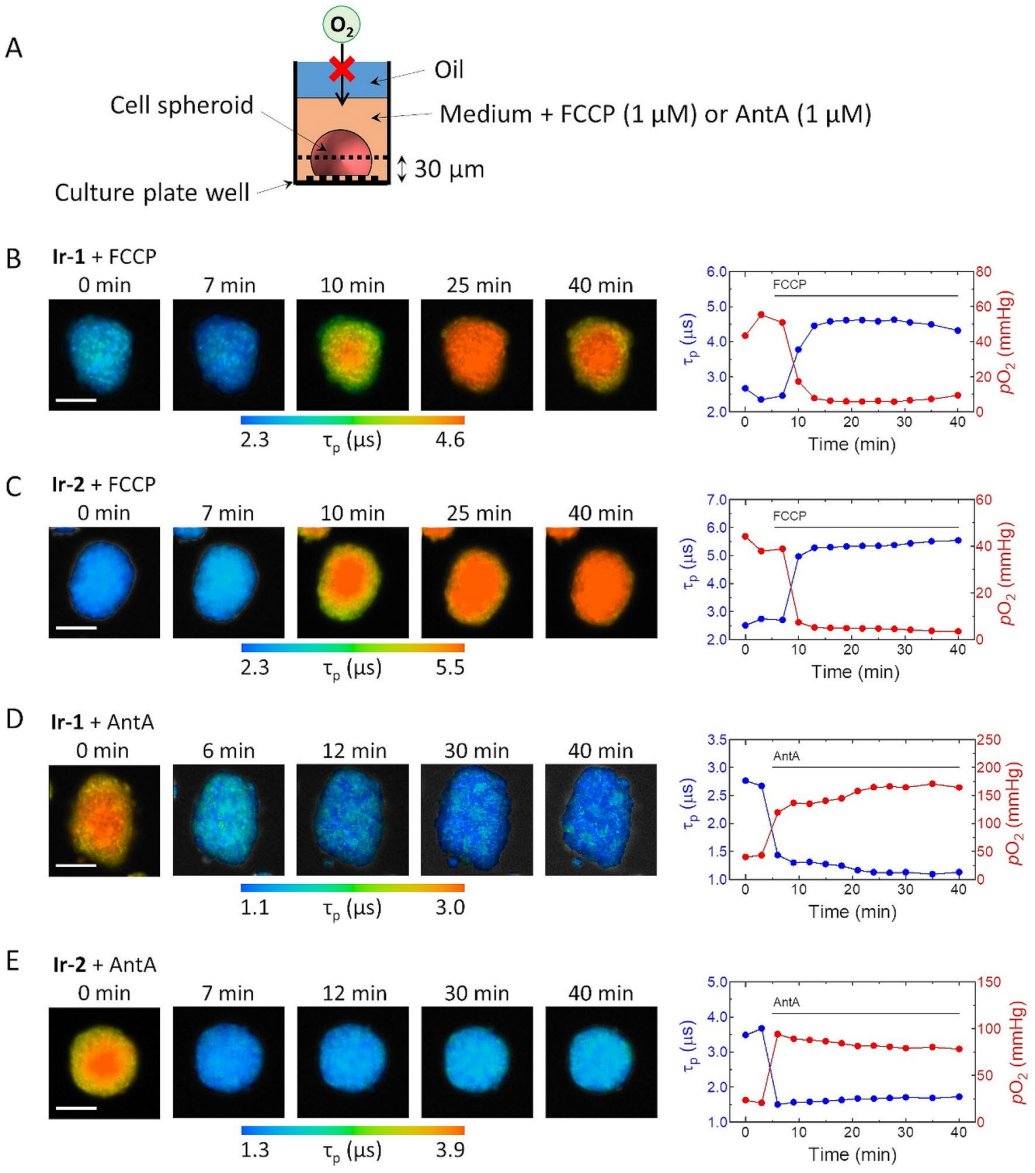
Imaging of the oxygen status of an HT-29 cell spheroid upon metabolic stimulation with FCCP or AntA. (**A**) Schematic representation of a spheroid in media containing FCCP or AntA. PLIM images were taken at 30 μm from the bottom after oil sealing. (**B**,**C**) Variation in PLIM images (left), and their average phosphorescence lifetime and *p*O_2_ (right) of an HT-29 cell spheroid stained with (**B**) **Ir-1** and (**C**) **Ir-2** by metabolic stimulation with FCCP (1 μM) at 5 min and 6 min, respectively, and (**D**) **Ir-1** and (**E**) **Ir-2** by metabolic stimulation with AntA (1 μM) at 5 min. Scale bar: 100 µm.

We next investigated whether **Ir-1** and **Ir-2** can image changes in the oxygen status of spheroids caused by metabolic stimulus. We used FCCP (carbonyl cyanide *p*-(trifluoromethoxy)phenylhydrazone) and AntA to alter the oxygen status of HT-29 spheroids by stimulating metabolic processes; FCCP is an uncoupler of oxidative phosphorylation in mitochondria that disrupts ATP synthesis by transporting protons across the membrane and thus increases the O_2_ consumption rate. AntA is known to inhibit the mitochondrial electron transport chain from cytochrome *b* to cytochrome *c*1 and suppress O_2_ consumption. Metabolic stimulation of HT-29 cell spheroids by FCCP and AntA significantly changed the PLIM images (Fig. 4). The lifetime images of the spheroid plane ~ 30 μm from the bottom (Fig. 4B,C) demonstrated that upon addition of FCCP to the medium, the lifetimes of both probes quickly increased within ~ 10 min, especially in the core. The oxygen partial pressures calculated from the lifetimes based on the calibration in Fig. 2 indicate that the *p*O_2_ decreased sharply to less than 20 mmHg by increased O_2_ consumption, and then it slowly recovered. Conversely, the lifetimes of both probes rapidly dropped within 10 min upon metabolic stimulation using AntA (Fig. 4D,E), corresponding to the increase in *p*O_2_ by suppression of O_2_ consumption. The *p*O_2_ changes of spheroids reflected the metabolic effects of FCCP and AntA, demonstrating that **Ir-1** and **Ir-2** can be used to track the oxygen status of cell spheroids. These results confirmed the high penetrating ability of **Ir-1** and **Ir-2** into spheroids and their feasibility as tissue O_2_ probes.

### In vivo O_2_ imaging of hepatic tissues

**Figure 5:**
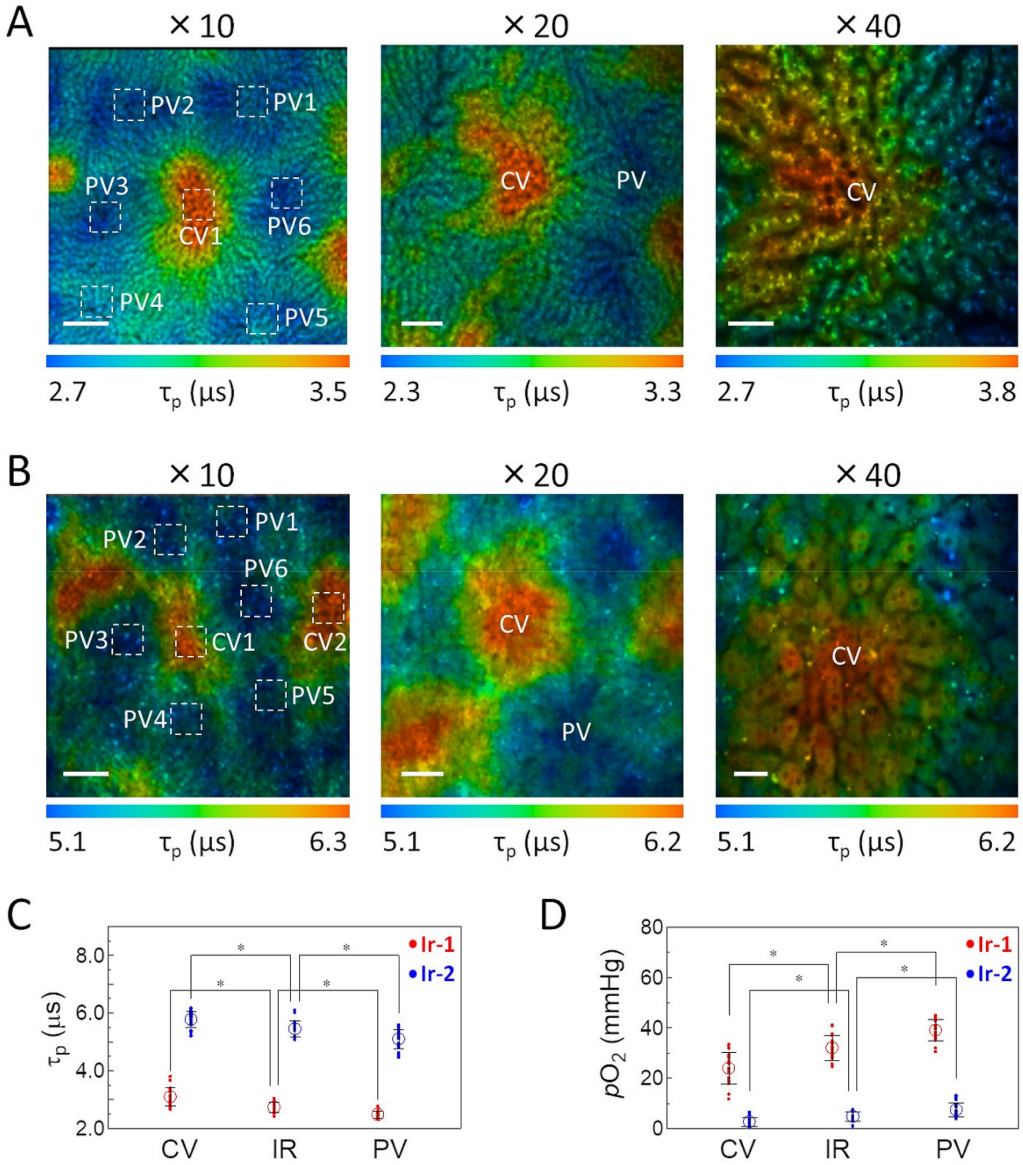
PLIM images of the hepatic surface of a mouse administered (**A**) **Ir-1** and (**B**) **Ir-2**. The color bar indicates phosphorescence lifetime in μs. Left images: 10 × objective lens, center images: 20 × objective lens, right images: 40 × objective lens. Scale bar: 200 µm (left images), 100 µm (center images), and 50 µm (right images). CV: central vein, PV: portal vein. (**C, D**) Phosphorescence lifetime of **Ir-1** and **Ir-2** and *p*O_2_ in hepatic lobules. Average of phosphorescence lifetime and *p*O_2_ are shown. *N* = 23 ROIs for CV, 23 for IR (intermediate region between CV and PV), and 24 for PV in 5 mice administered **Ir-1**. *N* = 21 ROIs for CV, 23 for MR, and 27 for PV in 5 mice administered **Ir-2**. *p value < 0.01 by 2-tailed unpaired *t* test. Error bar: S.D.

Since we confirmed that **Ir-1** and **Ir-2** can be efficiently taken up into spheroids and evaluate the oxygen status, we next attempted to visualize the oxygen gradient of hepatic lobules by in vivo PLIM measurements with **Ir-1** or **Ir-2** as a probe. Each probe (250 nmol in 25 μL dimethyl sulfoxide, diluted using PBS to 175 μL) was administered intravenously to anesthetized mice. Approximately 10 min after probe administration, the abdomen was opened to expose the liver, and the phosphorescence lifetime images of hepatic tissues (~ 10 μm from the surface of the liver) were measured around the central vein (CV) using different magnifications. Both probes gave clear PLIM images of hepatic tissues with cellular-level resolution (Fig. 5). Here, the areas that appear black are the regions where the emission intensity from the probe is extremely low, and these mainly correspond to the nucleus and sinusoid that have low probe uptake. It can be seen from the PLIM images measured with a 40 × objective lens that **Ir-1** and **Ir-2** are internalized into hepatocytes that line up along sinusoids. The arrangement of hepatocytes and lifetime distribution indicate that these images correspond to a hexagonal hepatic lobule (Fig. 1B) and that CV exists in the area with the longest lifetime (displayed in orange). The region surrounding the CV is considered to be the portal vein (PV) because it has a much shorter lifetime, i.e. much higher oxygen level. The direction of blood flow seen in the video image of hepatic tissues (Video S1) also indicates that the phosphorescence lifetime in the vicinity of CV is longer than that in the surrounding PV. PLIM images obtained with **Ir-1** and **Ir-2** show a similar lifetime gradient that increases from the PV to CV.

To quantify the O_2_ levels in specific locations in the lobules from the phosphorescence lifetimes, we performed calibration of the phosphorescence lifetimes using an AML 12 (alpha mouse liver 12) cell line that was established from mouse hepatocytes (Fig. S6). Using the τ_p_^0^ and *k*_q_ of **Ir-1** and **Ir-2** as determined in a monolayer of AML 12 cells, we evaluated the average *p*O_2_ of the regions of interest (ROIs) that can be attributed to CV, PV, and the intermediate region (IR); the phosphorescence lifetime of **Ir-1** gave reasonable O_2_ levels (24 ± 6.1 mmHg for CV, 32 ± 4.9 mmHg for IR, and 39 ± 4.2 mmHg for PV), whereas **Ir-2** exhibited much lower O_2_ levels (3 ± 1.8 mmHg for CV, 5 ± 1.8 mmHg for IR, and 7 ± 2.7 mmHg for PV) (Table 2). These results indicated that PLIM images obtained with **Ir-1** and **Ir-2** clearly visualized the oxygen gradient from PV to CV. However, the O_2_ levels derived from the phosphorescence lifetimes differed between **Ir-1** and **Ir-2**.

**Table 2:** Phosphorescence lifetimes ($\tau_{{\text{p}}}$) of **Ir-1** and **Ir-2** at around CV, IR, and PV in hepatic lobules and the *p*O_2_ values calculated from the lifetime.

Area	τ_p_ (μs)		*p*O_2_ (mmHg)	
	**Ir-1**	**Ir-2**	**Ir-1**	**Ir-2**
CV	3.09 ± 0.31	5.78 ± 0.28	24 ± 6.1	3 ± 1.8
IR	2.73 ± 0.18	5.45 ± 0.27	32 ± 4.9	5 ± 1.8
PV	2.48 ± 0.14	5.10 ± 0.33	39 ± 4.2	7 ± 2.7

*N* = 23 ROIs for CV, 23 for IR, and 24 for PV in 5 mice administered **Ir-1**. *N* = 21 ROIs for CV, 23 for IR, and 27 for PV in 5 mice administered **Ir-2.**

To confirm that **Ir-1** and **Ir-2** retain their spectral properties in hepatic tissues after systemic administration, we measured the emission spectra of the livers of living mice. Each probe molecule (250 nmol) was administered through the tail vein, and the emission images and spectra of livers were taken using a fluorescence microscope with an excitation filter of 450–500 nm and emission filter of > 532 nm (Fig. S7). The observed emission spectra with maxima at 619 nm for **Ir-1** and 607 nm for **Ir-2** were slightly red-shifted compared with those in MeCN, but they were in good agreement with those in AML 12 cells, demonstrating that these probes maintain their spectral properties in hepatic tissue.

**Figure 6:**
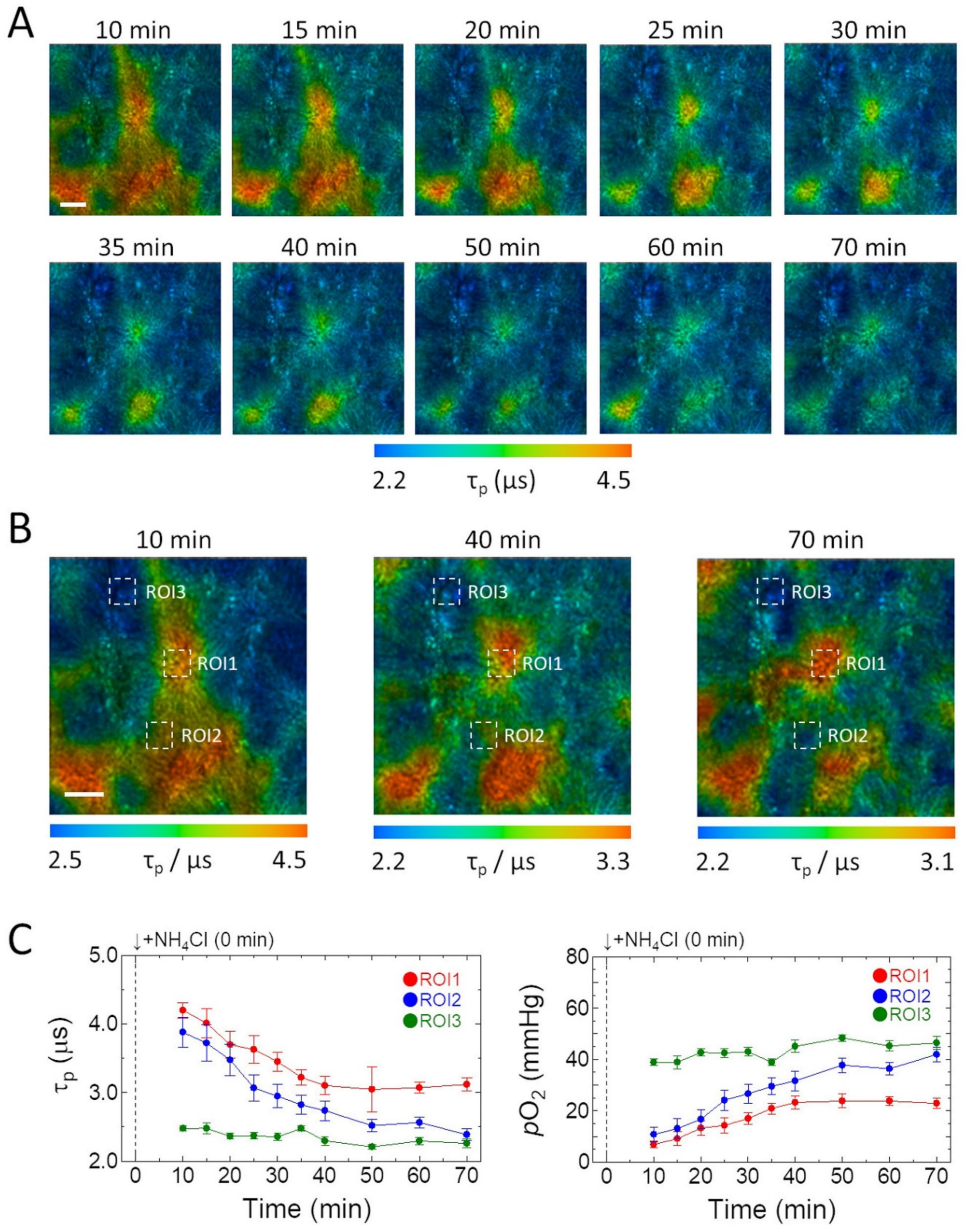
(**A,B**) Variation of PLIM images of hepatic lobules after administration of NH_4_Cl following **Ir-1** injection into the tail vein of a mouse. Scale bar: 200 µm. (**C,D**) Variation of average phosphorescence lifetime and *p*O_2_ of ROIs in the pericentral region (ROI1) and periportal regions (ROI2 and ROI3) shown in **B**. Error bar: S.D.

The reason why **Ir-2** gave an abnormally low O_2_ level seems to be related to the function of the liver to metabolize xenobiotics. So, to clarify how clearance of harmful metabolic byproducts and detoxification of xenobiotics affect hepatic O_2_ levels, we investigated the detoxification of ammonia in the liver, which has the function of converting toxic ammonia to urea by the urea cycle or to glutamine by glutamine synthesis in hepatocytes. With increased metabolic activity of the liver, oxygen consumption should be enhanced to produce ATP. We intravenously administered NH_4_Cl (0.13 g/kg) to anesthetized mice at ~ 30 min after 250 nmol **Ir-1** injection into the tail vein. PLIM images of hepatic lobules measured 10 min after the administration of NH_4_Cl exhibited a marked increase in the phosphorescence lifetime of **Ir-1** (Fig. 6A,B). The average phosphorescence lifetimes (τ_p_) and *p*O_2_ of different ROIs in the pericentral region (ROI1) and periportal regions (ROI2 and ROI3) showed that the lifetimes decreased over time, and the O_2_ levels recovered to the original levels in approximately 1 h.

To clarify the difference in in vivo probe performance between **Ir-1** and **Ir-2**, we performed O_2_ imaging experiments on liver tissues using another Ir(III) complex, (btp)_2_Ir(acac-2OH) (**Ir-3**), which has a similar structure to **Ir-1** (Fig. S8A). The $\tau_{{\text{p}}}^{0}$ and *k*_q_ of **Ir-3** in AML 12 cells were determined to be 4.80 µs and 4.82 × 10^3^ mmHg^−1^ s^−1^. The PLIM image (Fig. S8B) in the hepatic lobule of an **Ir-3**-administered mouse exhibited a clear lifetime gradient and gave an average *p*O_2_ of 16 ± 3.2 mmHg in CV, 21 ± 3.4 mmHg in IR, and 24 ± 3.8 mmHg in PV (Fig. S8C). The hepatic O_2_ levels obtained with **Ir-3** were close to those obtained with **Ir-1**, although **Ir-3** gave somewhat smaller *p*O_2_ values.

In contrast to the results in the liver tissue, **Ir-1** and **Ir-2** gave similar O_2_ tensions in the kidney (Fig. S9). Intravenously administered probes rapidly migrated from the vasculature into the proximal tubule cells and were hardly excreted in the urinary space during the observation period. The average O_2_ tension of tubular cells in each image was derived using calibration lines obtained for human kidney 2 (HK-2) cells to be 45 ± 9.8 mmHg for **Ir-1** and 36 ± 4.3 mmHg for **Ir-2** (Fig. S10).

## Discussion

We first verified that **Ir-1** and **Ir-2** exhibit desired photophysical properties as O_2_ probes in cells and cell spheroids. Although the τ_p_^0^ values (5–10 μs) of **Ir-1** and **Ir-2** are much smaller than those of Pt(II)- and Pd(II)-porphyrins, their oxygen sensitivity is sufficiently high due to their large *k*_q_ values. In addition, the moderately long lifetimes of the Ir(III) complexes have the advantage of increasing the counting efficiency in PLIM measurements and thus shortening the image acquisition time.

One key issue in applying small-molecule intracellular oxygen probes for in vivo O_2_ measurements is the method of lifetime calibration. We quantified the tissue *p*O_2_ based on the *k*_q_ and τ_p_^0^ determined using a cell line that is the same as or close to the cell type in the target organ in which the probe is accumulated. The O_2_ quenching rate constants in HT-29 and AML 12 cells were reduced by an order of magnitude compared with those in solution (see Table 1). Since lipophilic **Ir-1** and **Ir-2** are likely to accumulate in organelle membranes after they pass through the plasma membranes of living cells, the much smaller *k*_q_ values in cells can be attributed, at least in part, to the decrease in the diffusion rates of O_2_ and Ir(III) complexes in organelle membranes. Further reductions in *k*_q_ values can be caused by the binding of excited probe molecules to proteins in the organelle membrane in which they are incorporated. The τ_p_^0^ values of **Ir-1** and **Ir-2** in cells were decreased by 10–15% compared with those in MeCN. One possible reason lies in the concentration quenching in organelle membranes. The τ_p_^0^ of **Ir-1** partitioned into DMPC liposomes tended to shorten with increasing probe concentration in solution (Table S1), suggesting that concentration quenching may have occurred. Cross-sensitivity to endogenous species other than O_2_ cannot be ruled out, but its contribution appears to be relatively small because the τ_p_^0^ values of **Ir-1** and **Ir-2** in cells are close to those in MeCN. It was also confirmed that phosphorescence lifetime is almost independent of pH and the presence of glutathione under physiological conditions (Tables S2 and S3).

One of the major advantages of **Ir-1** and **Ir-2** is their extremely high cellular uptake efficiencies. The O_2_ imaging experiments using HT-29 cell spheroids (Fig. 3) demonstrated that **Ir-1** and **Ir-2** are efficiently internalized into the spheroids with a diameter of ~ 200 μm. The penetrative abilities of some molecular O_2_ probes into cell spheroids reported so far are compared in Table S4. These probes include glucose conjugates of Pt(II)-*meso*-tetrakis-(pentafluorophenyl)porphyrin^20^, Pt(II)-5, 10, 15, 20-tetrakis-(4-carboxyphenyl)porphyrin^36^, a Pt(II) complex bearing a cyclometalating 3-di(2-pyridyl)benzene-based moiety^35^, and click-assembled oxygen-sensing nanoconjugates with Pd(II) tetracarboxytetrabenzoporphyrin as a phosphorescent core^24^. Comparing each probe for its concentration in culture medium and the incubation time required for observation of clear luminescence images, it can be seen that **Ir-1** and **Ir-2** have excellent cell permeability. Furthermore, PLIM measurements with these probes made it possible to track oxygen levels and distributions in spheroids. These properties are very useful for investigating the oxygen status in tissues.

The oxygen gradient in the hepatic lobule has been demonstrated by applying the hypoxia marker 2-nitroimidazole to liver tissue sections^53^. High-resolution visualization of hepatic oxygen distribution in vivo has been performed by Paxian et al.^44^ by intravital emission microscopy using [Ru(phen)_3_]^2+^ (Tris(1,10-phenanthroline)ruthenium(II)) as an O_2_ probe. They observed a continuous increase in emission intensity of [Ru(phen)_3_]^2+^ from periportal to pericentral regions in rat liver, implying an O_2_ gradient within the liver tissue. However, the emission intensity depends on the probe distribution in the tissue, so that intensity-based measurements cannot assess the precise *p*O_2_ and O_2_ gradient. As for hepatic tissue oxygen levels, Kietzmann and Jungermann^54,55^ have reported the *p*O_2_ to be 60–65 mmHg in the periportal blood and 30–35 mmHg in the perivenous blood, and they suggested the *p*O_2_ level to be approximately 15 mmHg lower in periportal and perivenous cells. Tsukada and Suematsu^56^ have investigated the average *p*O_2_ in hepatic microcirculation by phosphorescence lifetime measurements of BSA (bovine serum albumin)-bound Pd-TCPP (Pd(II)-meso-tetra(4-carboxyphenyl)porphine) in hepatic tissues of mice. They obtained an average *p*O_2_ of 59.8 mmHg in portal vessels and 38.9 mmHg in central venules. Our PLIM measurements with **Ir-1** showed that the average *p*O_2_ of hepatocytes near PV and CV were 39 ± 4.2 mmHg and 24 ± 6.1 mmHg, respectively. Although the hepatic oxygen levels obtained in our study are somewhat lower than those reported for intravascular *p*O_2_, these are considered to be reasonable values given the oxygen levels to which hepatocytes are exposed. To the best of our knowledge, the PLIM images shown in Fig. 5 are the first to visualize the oxygen concentration gradient in hepatic lobules in vivo using an intracellular O_2_ probe.

Imaging the spatiotemporal *p*O_2_ changes within the liver microarchitecture provides useful information about the oxygen response and dynamics of the liver at the cellular level during physiological stimulation. Our PLIM measurements with **Ir-1** showed that intravenous administration of NH_4_Cl resulted in a rapid decrease in hepatic tissue O_2_ levels, and its recovery rate depended on the position in the lobule (Fig. 6). Hepatocytes are known to display considerable functional differences depending on their position along the porto-central axis of the liver lobule, and ammonia detoxification in the liver occurs by two pathways: consumption by urea synthesis in the periportal area and removal by glutamine synthesis in perivenous area. Since these processes require O_2_ consumption, ammonia stimulation may produce hypoxic regions in the hepatic lobules depending on the O_2_ consumption rate of hepatocytes.

In HT-29 cell spheroids, **Ir-1** and **Ir-2** showed almost the same O_2_ levels and distribution, whereas in hepatic tissues of living mice, **Ir-2** gave much lower oxygen levels: 7 ± 2.7 mmHg for the periportal region and 3 ± 1.8 mmHg for the perivenous region (Table 2). In kidneys, on the other hand, both probes accumulated in tubular cells and showed similar oxygen levels: 45 ± 9.8 mmHg for **Ir-1** and 36 ± 4.3 mmHg for **Ir-2** (Fig. S9). These results suggest that the administration of **Ir-2** to mice causes hypoxia in hepatic tissues due to some specific metabolic processes in the liver. Since spectral measurements (Fig. S7) revealed that the luminescent properties of **Ir-1** and **Ir-2** were maintained in liver tissue, it is likely that **Ir-2** is excreted in the bile through solubilization processes that do not affect the luminescent properties such as glucuronidation at the OH group. The luminescence spectrum of the extract from bile 6 h after probe administration showed that **Ir-1** was excreted through the bile duct along with bile (Fig. S11). Considering that **Ir-3**, which has the same acac moiety as **Ir-1**, gave similar liver tissue O_2_ levels as **Ir-1**, the lipophilic Tris-ligand structure of **Ir-2** may be related to its metabolism in the liver.

## Conclusions

Small-molecule O_2_ probes, **Ir-1** and **Ir-2**, were efficiently taken up into cells when added to the culture media of monolayer cells and cell spheroids, and this enabled reversible *p*O_2_ measurements in the cells. PLIM measurements of mouse livers following intravenous administration of **Ir-1** or **Ir-2** allowed high-resolution O_2_ imaging of hepatic tissues, exhibiting an O_2_ gradient from the pericentral to periportal regions in hepatic lobules. The phosphorescence lifetime of **Ir-1** gave reasonable hepatic O_2_ levels after calibrating the lifetime using cultured AML 12 cells. Furthermore, **Ir-1** allowed visualization of the *p*O_2_ changes in hepatic tissues stimulated by ammonia. However, **Ir-2** gave a much lower *p*O_2_ compared with **Ir-1**, which may be due to the toxicity of **Ir-2**, which promoted detoxification in the liver. These results reveal that Ir(III) complexes allow imaging of spatiotemporal changes in oxygen levels within the tissue microarchitecture in vivo, but some complexes may influence oxygen consumption in the liver when used for oxygen imaging of hepatic tissues.

## Methods

### Materials

The Ir(III) complexes used in this study were synthesized and characterized according to the methods described in the Supplementary Information.

Acetonitrile (MeCN; Kanto Chemical, spectroscopic grade) and NH_4_Cl (Kanto Chemical, special grade) were used as received. Antimycin A from *Streptomyces* sp. (Ant A) and carbonyl cyanide 4-(trifluoromethoxy) phenylhydrozone (FCCP, 98%) were purchased from Sigma-Aldrich. Cell Counting Kit-8 (CCK-8) was purchased from Dojindo Laboratories.

^1^H-NMR spectra were recorded with a JEOL JNM-ECS400 (400 MHz) spectrometer in DMSO-*d*_6_. ^1^H-NMR chemical shifts were referenced to tetramethylsilane. The apparent resonance multiplicity was described as s (singlet), d (doublet), dd (double doublet), t (triplet), and m (multiplet). ESI–MS spectra were recorded on an Applied Biosystems API 2000 mass spectrometer.

### Photophysical properties in solution

Absorption spectra were recorded on a UV/Vis spectrophotometer (Ubest-V550, JASCO). Emission spectra were measured with a system consisting of a monochromatized Xe arc lamp, a sample holder, and a photonic multichannel analyzer (C10027-01, Hamamatsu Photonics), and were fully corrected for spectral sensitivity between 200–950 nm. Phosphorescence lifetimes in solution were measured with a lifetime measurement system (Quantaurus-Tau C11367G, Hamamatsu Photonics) based on the time-correlated single photon counting (TCSPC) method. Phosphorescence quantum yield was measured with an absolute photoluminescence quantum yield measurement system (C9920-02, Hamamatsu Photonics) that consisted of a Xe arc lamp, a monochromator, an integrating sphere, a multichannel detector, and a personal computer^57^.

### Cell and cell spheroid cultures

Human colorectal adenocarcinoma (HT-29) cells and HeLa cells were purchased from the American Type Culture Collection (ATCC). Alpha mouse liver 12 cells (AML12 cells) were kindly provided by Prof. T. Inagaki of the Laboratory of Epigenetics and Metabolism, IMCR, Gunma University. Human kidney 2 cells (HK-2 cells) were kindly provided by Prof. M. Nangaku of the Graduate School of Medicine, the University of Tokyo. HT-29 cells were incubated in McCoy’s 5A medium (Gibco) containing 10% fetal bovine serum (FBS), penicillin (50 units/mL), and streptomycin (50 µg/mL). AML12 cells and HK-2 cells were incubated at 37 °C in a mixture of Dulbecco’s Modified Eagle’s Medium (DMEM) and Ham’s F-12 Nutrient Mixture containing 10% FBS, penicillin (50 units/mL), and streptomycin (50 µg/mL). All cells were grown in a humidified 5% CO_2_ enriched atmosphere at 37 °C. In PLIM imaging, all cells were cultured on glass-based dishes, and the medium was changed to McCoy’s 5A or DMEM/F-12 without phenol red and FBS prior to the measurements.

HT-29 cell spheroids were prepared using a low attachment 96-well NanoCulture Plate (NCP; NCP-LH96, ORGANOGENICS) with a micro-honeycomb pattern on the film that comprises the bottom of the plate. McCoy’s 5A medium (100 μL) containing 10% FBS, penicillin (50 units/mL), and streptomycin (50 µg/mL) was added to each well in the NCP, and the NCP was centrifuged at 10 × 100 rpm for 5 min to remove the bubbles in the media in the wells. Then HT-29 cells (1.0 × 10^5^ cells/mL) suspended in 100 μL McCoy’s 5A medium were added to each well in the NCP. The cells were incubated in the NCP at 37 °C, 5% CO_2_ for 4 days to form the HT-29 cell spheroids.

### PLIM imaging of cultured cells

PLIM images were measured using an inverted fluorescence microscope (IX73, Olympus) equipped with a picosecond diode laser (BDL-SMC, Becker & Hickl; wavelength: 488 nm, pulse width: 40–90 ps, repetition rate: 50 MHz) and a confocal scanning system (DCS-120, Becker & Hickl). A 4 × objective (UPlanSApo; NA 0.16, Olympus), 10 × objective (UPlanSApo; NA 0.40, Olympus), 20 × objective (UCPlanFLN; NA 0.70, Olympus), 40 × oil objective (UPlanFLN; NA 1.30, Olympus), or 100 × oil objective (UPlanSApo; NA 1.40, Olympus) were used for PLIM imaging. The emission, as guided through a 1 mm pinhole, was collected through a longpass filter and detected with a hybrid detector module (HPM-100–40, Becker & Hickl). Time-resolved emission measurements were performed with a time-correlated single photon counting (TCSPC) system (Simple-Tau-150-DX, Becker & Hickl). The emission signals were acquired at a resolution of 128 × 128 pixels, and the decay curve in each pixel was fitted to single- or double-exponential decay function. In the case of double-exponential decay, the amplitude average lifetime was used for Stern–Volmer analyses. PLIM images were analyzed with SPCImage data analysis software (Becker and Hickl). In PLIM imaging of cell spheroids, HT-29 sell spheroids were incubated in the McCoy’s 5A medium containing a final concentration of 500 nM **Ir-1** or **Ir-2** for 24 h at 37 °C, 5% CO_2_ prior to the measurements.

### In vivo PLIM measurements

All protocols for animal experiments were approved by the Ethical Committee on Animal Experiments of Gunma University (18-018), and all animal experiments were conducted in accordance with the institutional guidelines. In vivo PLIM measurements were carried out using six- to eight-week-old *Balb/c* male mice (CLEA Japan). After general anesthesia, probe solution (200 µL) containing 250 nmol Ir(III) complex in 10% DMSO/saline was injected into the tail vein, and the liver or kidney was exposed for ~ 10 min before PLIM experiments.

### Calibration of the phosphorescence lifetime

The oxygen partial pressure in cells was obtained from the phosphorescence lifetime based on a calibration using cultured cells. HT-29, AML12, or HK-2 cells were seeded on glass-based dishes and stained with **Ir-1** or **Ir-2** (500 nM, 2 h). PLIM images were acquired for the stained cells at 21%, 15%, 10%, 5%, and 0% O_2_ in an incubator at 37 °C. Here, in the 5–21% O_2_ experiments, cells were incubated with Ant A (10 µM, > 0.5 h) to block cellular respiration, and in the 0% O_2_ experiments, fresh medium containing Na_2_SO_3_ (500 mM) was used to remove existing O_2_ in the medium. The average phosphorescence lifetimes of the PLIM images taken under different *p*O_2_ were plotted according to the Stern–Volmer equation, τ_p_^0^/τ_p_ = 1 + *k*_q_τ_p_^0^*p*O_2_, to determine the bimolecular quenching rate constant, *k*_q_. The *p*O_2_ of cells, spheroids, and tissues was obtained from the phosphorescence lifetime (τ_p_) using the *k*_q_ and τ_p_^0^.

### Subcellular localization of probes

HeLa cells were cultured in DMEM (Gibco) with 10% FBS, penicillin (50 units/mL), and streptomycin (50 μg/mL), and were grown under a 5% CO_2_ atmosphere at 37 °C. The cells were seeded into glass bottom imaging dishes (Greiner) and allowed to adhere for 24 h, incubated with **Ir-1** (500 nM) or **Ir-2** (1 μM) for 2 h, washed 3 times with DMEM, and then the medium was replaced with DMEM (FluoroBrite, Gibco) without FBS. Luminescence microscopic images were obtained with an inverted microscope (IX71, Olympus) equipped with an electron multiplying CCD camera (Evolve 512, PHOTOMETRICS) driven by MetaMorph software.

### Evaluation of cytotoxicity using CCK-8 assay

HT-29 cells (2.5 × 10^4^ cells/well) were seeded into a 96-well flat bottom plates (Greiner) for 48 h. The cells were incubated with various concentrations of each probe for 24 h at 37 °C under a 5% CO_2_ atmosphere. The medium was removed, and the cells were washed gently with McCoy’s 5A medium without phenol red. Cell Counting Kit-8 reagent (CCK-8, Dojindo) was added to each well, and incubation was continued for 1 h^58^. The absorbance at 450 nm of each well referenced at 650 nm was recorded using a microplate reader (Infinite 200 PRO, Tecan). Cell viability (% of control) was evaluated as (*A*_sample_ − *A*_blank_)/(*A*_control_ − *A*_blank_) × 100, where *A*_sample_ is the absorbance of cells exposed to the probe, *A*_control_ is the absorbance of cells without probe, and *A*_blank_ is the absorbance of wells without cells.

### Mitochondrial membrane potential

HT-29 cells (3.0 × 10^4^ cells/well) were seeded into a 96-well flat bottom plates (Greiner) for 48 h. The cells were stained with **Ir-1** or **Ir-2** (1 μM, 2 h). The medium was removed, and the cells were washed gently with McCoy’s 5A medium without phenol red. 5,5′,6,6′-tetrachloro-1,1′-3,3′-tetraethyl-benzimidazolylcarbocyanine iodide (JC-1, Thermo Fisher Scientific) (5 μM) was added to each well, and incubation was continued for 30 min. The medium was removed, and the cells were washed gently with McCoy’s 5A medium without phenol red. The emission intensities of JC-1 were measured using a microplate reader (Infinite 200 PRO, Tecan) equipped with a gas control module (GCM, Tecan). The excitation wavelength was 488 nm, and the monitor wavelength was 595 nm for Red emission (aggregate) and 540 nm for green emission (monomer).

## Supplementary information

Supplementary Information 1.

Supplementary Video.
