# Supplementary material for: In vivo O2 imaging in hepatic tissues by phosphorescence lifetime imaging microscopy using Ir(III) complexes as intracellular probes
Source: Sci Rep. 2020 Dec 3;10:21053. doi: 10.1038/s41598-020-76878-6 (PMC7713648; doi:10.1038/s41598-020-76878-6)
Supplement: Supplementary file 1 — Supplementary Information 1. [file 41598_2020_76878_MOESM1_ESM.docx]

Supplementary Information

***In vivo* O_2_ imaging in hepatic tissues by phosphorescence lifetime imaging microscopy using Ir(III) complexes as intracellular probes**

Kiichi Mizukami^1^, Ayaka Katano^1^, Shuichi Shiozaki^1^, Toshitada Yoshihara^1^*, Nobuhito Goda^2^ & Seiji Tobita^1^*

^1^ Department of Chemistry and Chemical Biology, School of Science and Technology, Gunma University, Kiryu, Gunma 376-8515, Japan

^2^ Department of Life Science and Medical BioScience, School of Advanced Science and Engineering, Waseda University, Wakamatsu-cho, Shinjuku-ku, Tokyo 162-8480, Japan

E-mail: tobita @gunma-u.ac.jp, yoshihara@gunma-u.ac.jp

This PDF file includes:

Figures S1 to S10

Table S1

Video movie

Materials and Methods

References


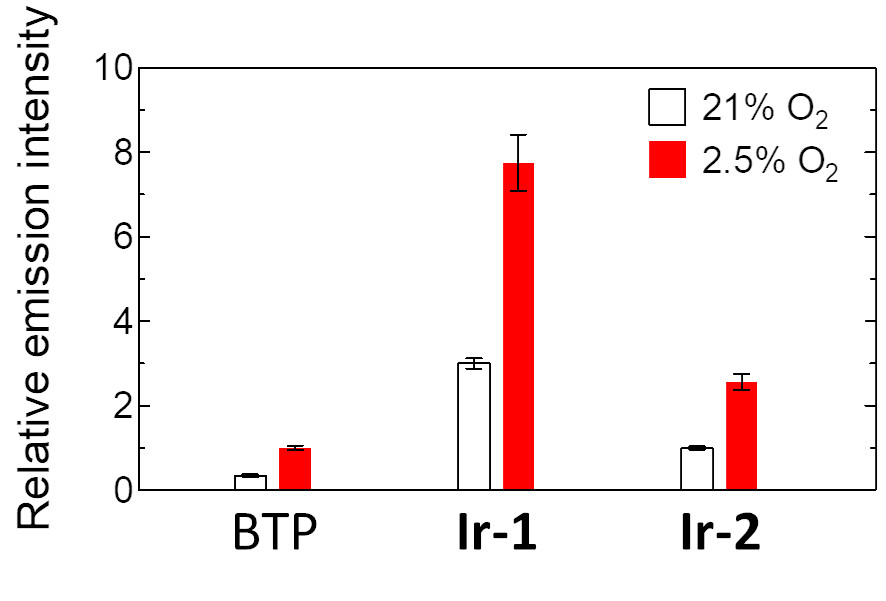


**Fig. S1** Relative emission intensities of each probe taken up into HT-29 cells incubated with BTP, **Ir-1** or **Ir-2** (5 μM, 2 h) at 37 °C. The emission intensities measured with a microplate reader (Infinite 200 PRO, Tecan) were corrected for the number of HT-29 cells in each well and the molar extinction coefficient at 488 nm in MeCN (4700, 5400, and 2800 M^-1^cm^-1^ for BTP, **Ir-1** and **Ir-2,** respectively). Excited at 488 nm. Monitored at 615 nm (BTP), 616 nm (**Ir-1**) and 601 nm (**Ir-2**). *N* = 3. Error bar: S.D.

**
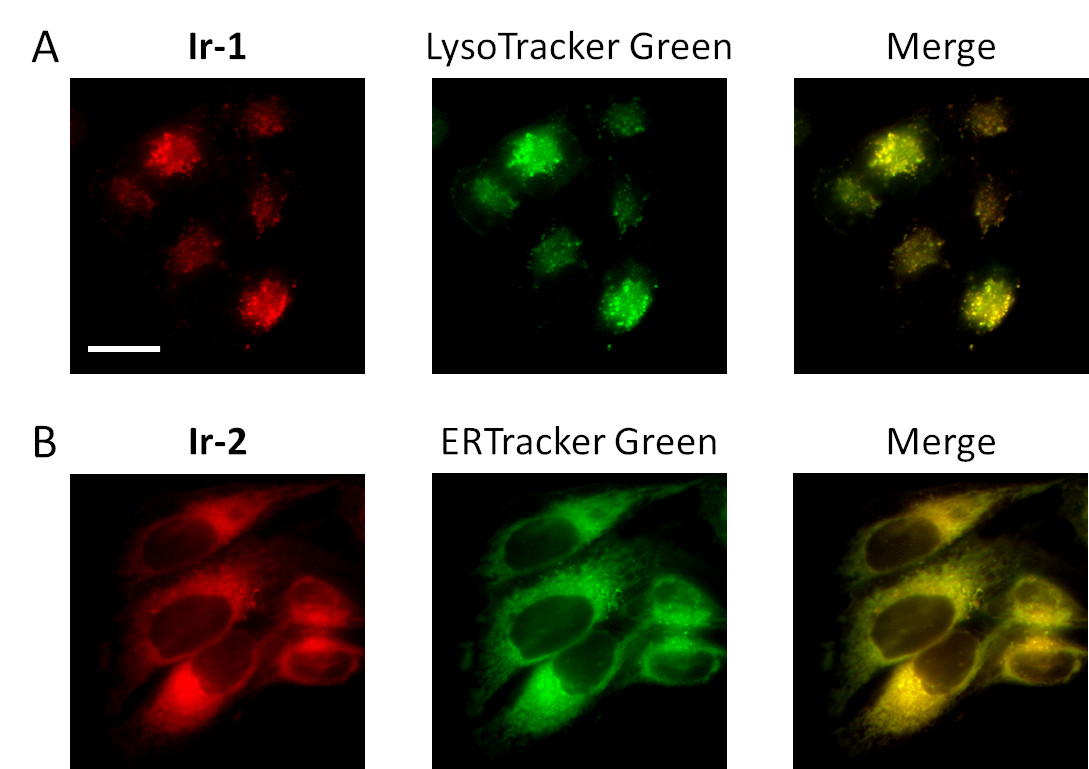
**

**Fig. S2** Subcellular localization of **Ir-1** (**A**) and **Ir-2** (**B**) in HeLa cells. The emission images of HeLa cells stained with **Ir-1** (**A**, left), **Ir-2** (**B**, left), organelle-selective markers (middle) and their overlay images (right). The Pearson’s correlation coefficients were calculated to be 0.96 for **Ir-1** and 0.98 for **Ir-2**. Cells was costained with **Ir-1** (500 nM, 2 h) and LysoTracker Green (200 nM, 20 min), and **Ir-2** (1 μM, 2 h) and ERTracker Green (200 nM, 20 min). Scale bar: 20 µm. *λ*_ex_: 400-440 nm, *λ*_em_: >590 nm (left). *λ*_ex_: 450-500 nm, *λ*_em_: 515-565 nm (middle).

**
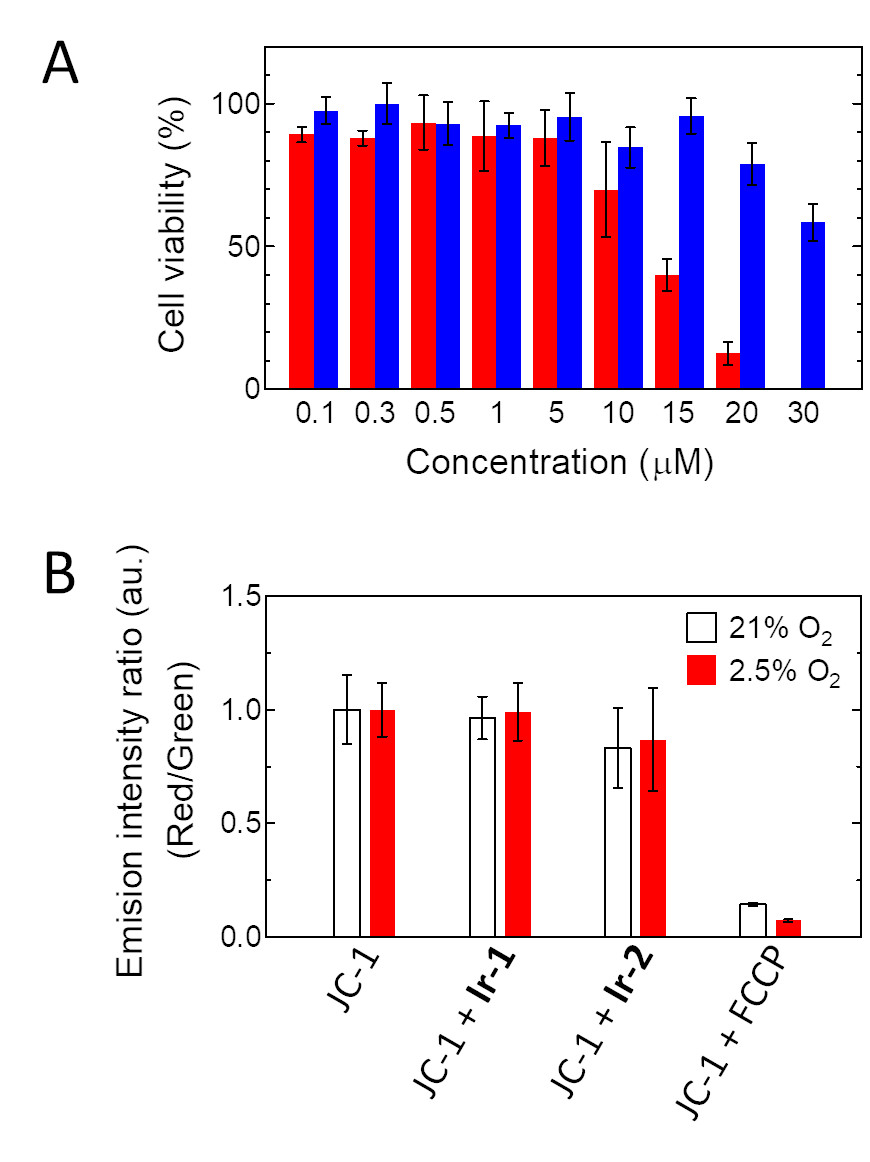
**

**Fig. S3** Assessment of the cytotoxicity of **Ir-1** and **Ir-2**. (**A**) Cell viability of HT-29 cells when incubated with various concentrations of **Ir-1** (red) and **Ir-2** (blue) for 24 h, evaluated by the WST assay. Error bar: S.D. (**B**) Emission intensity ratio (Red/Green) of JC-1 in HT-29 cells stained with **Ir-1**, **Ir-2** (1 μM, 2 h) and FCCP (10 μM, 1 h). *λ*_ex_: 488 nm, *λ*_em_: 595 nm (Red emission). *λ*_ex_: 488 nm, *λ*_em_: 540 nm (Green emission). *N* = 12. Error bar: S.D.


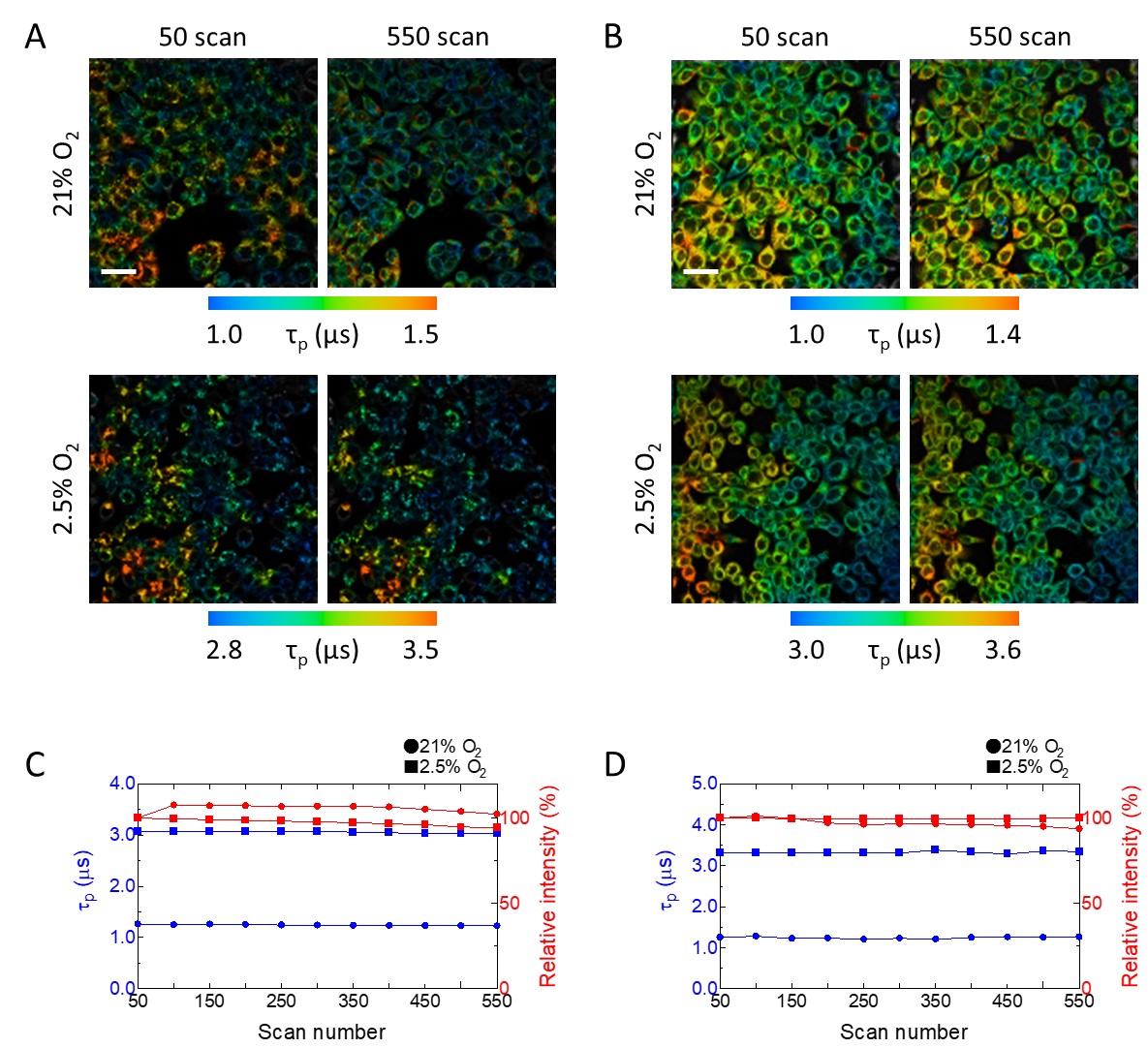


**Fig. S4** Photostability of **Ir-1** and **Ir-2** in 2D cultured HT-29 cells. (**A, B**) PLIM images of monolayered HT-29 cells stained with **Ir-1** (**A**) and **Ir-2** (**B**) acquired after 50 and 550 scans at 488 nm irradiation under 21% and 2.5% O_2_. (**C, D**) Variation of average phosphorescence lifetime (blue) and intensity (red) derived from the PLIM images of HT-29 cells stained with **Ir-1** (**C**) and **Ir-2** (**D**). Each image was acquired every 50 scans up to 550 scans. Probe concentration: 1 µM, incubation time: 2 h. Scale bar: 50 µm.


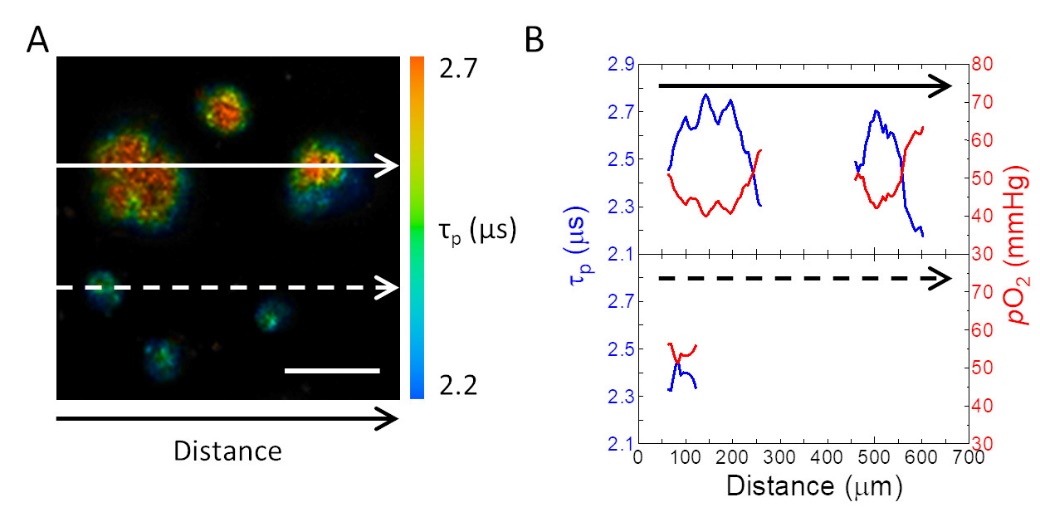


**Fig. S5** (**A**) PLIM images of HT-29 cell spheroids with different sizes stained with **Ir-1**. Scale bar: 100 µm. The emission image was taken at *z* = 0 µm. (**B**) Line profile of phosphorescence lifetime and *p*O_2_ in the HT-29 cell spheroids along the solid arrow (top) and dashed arrow (bottom) in **A**.


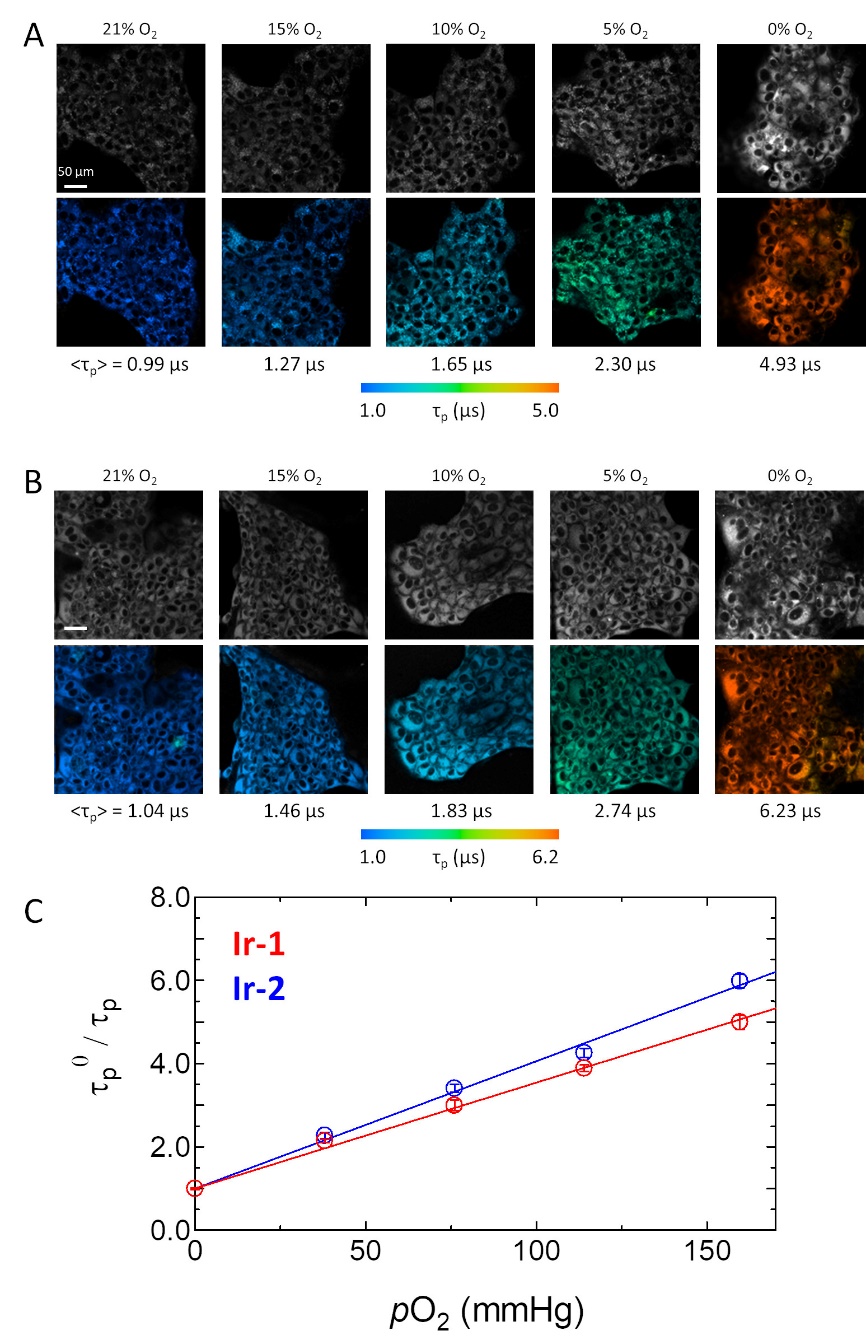


**Fig. S6** (**A**, **B**) Phosphorescence intensity (upper figures) and PLIM (lower figures) images of AML 12 cells stained with **Ir-1** (**A**) and **Ir-2** (**B**) under different oxygen partial pressures in an incubator. The average phosphorescence lifetime is shown under each image. Cells are treated with Ant A (5-21% O_2_) to suppress cell respiration and Na_2_SO_3_ (0% O_2_) to remove dissolved oxygen. Scale bar: 50 µm. (**C**) Stern-Volmer plots of $\tau_{p}^{0}$/$\tau_{p}$ as a function of oxygen partial pressure for **Ir-1** (red) and **Ir-2** (blue) taken up into AML 12 cells. The $\tau_{p}^{0}$ and *k*_q_ in AML 12 cells are 4.93 µs and 5.16×10^3^ mmHg^-1^s^-1^ for **Ir-1** and 6.23 µs and 4.91×10^3^ mmHg^-1^s^-1^ for **Ir-2**. Error bar: S.D.


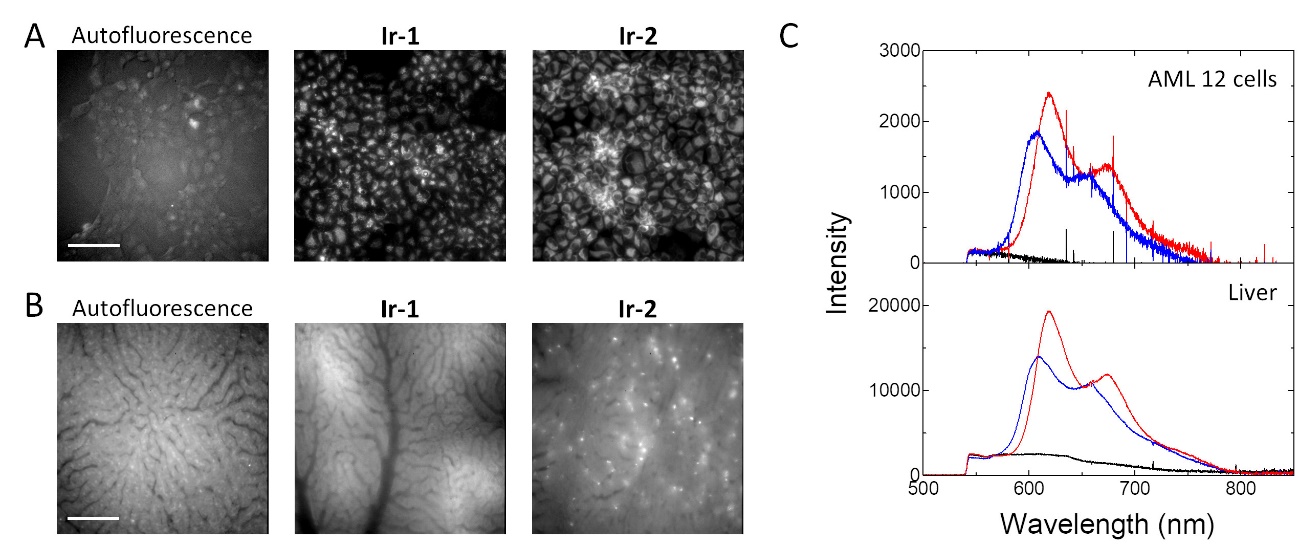


**Fig. S7** Emission images and spectra of AML 12 cells and liver surface of a living mouse.

(**A**) Autofluorescence of AML 12 cells (left), emission images of AML 12 cells incubated with **Ir-1** (middle) and **Ir-2** (right) (1 μM, 2 h). *λ*_ex_: 450-500 nm, *λ*_em_: >532 nm. Scale bar: 100 µm; (**B**) Emission intensity images of mouse liver with and without probe administration. Autofluorescence in the absence of probe (left), with administration of 250 nmol **Ir-1** (middle) and 250 nmol **Ir-2** (right). *λ*_ex_: 450-500 nm, *λ*_em_: >532 nm. Scale bar: 100 µm; (**C**) Emission spectra derived from the images in **A** (upper) and **B** (lower), respectively. Black: autofluorescence in the absence of probe, red: **Ir-1,** blue: **Ir-2**, *λ*_ex_: 450-500 nm.


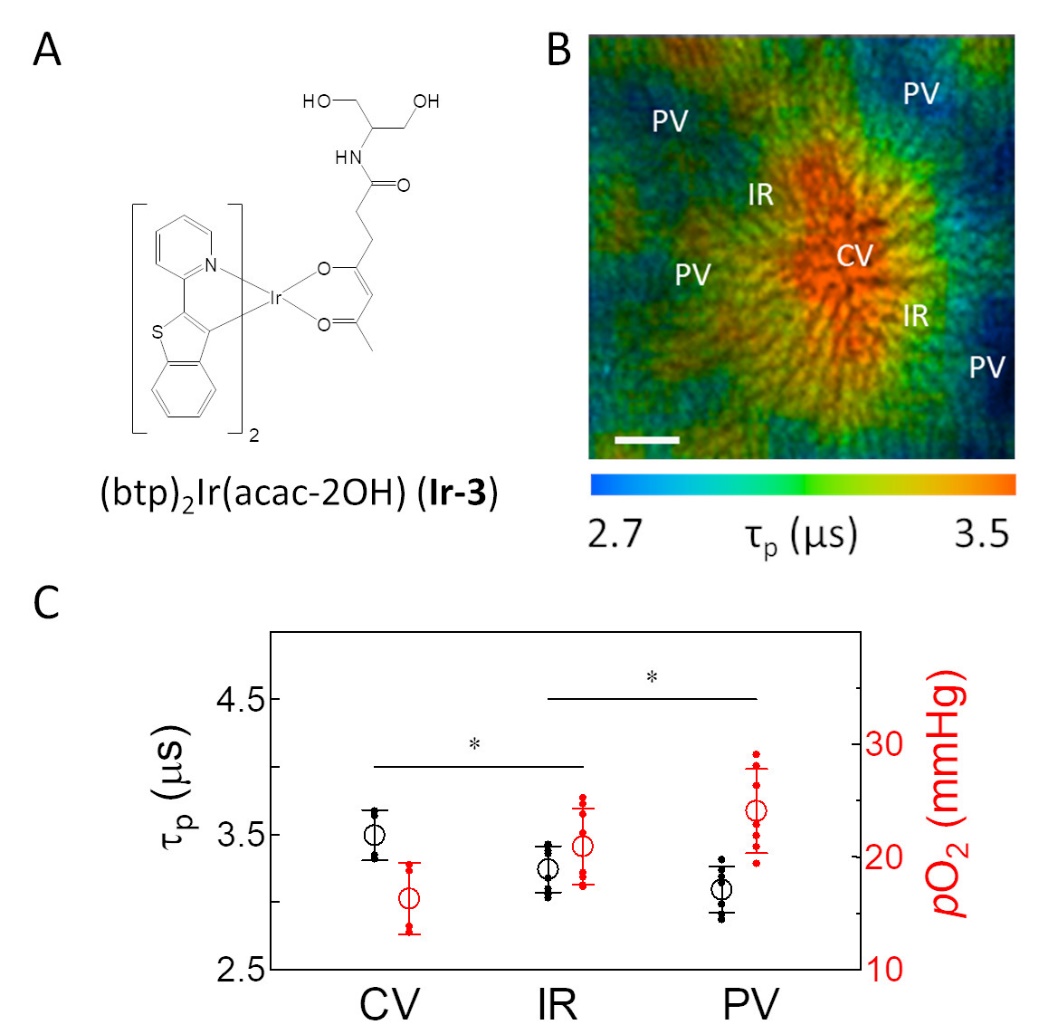


**Fig. S8** In vivo O_2_ imaging in hepatic tissues using (btp)_2_Ir(acac-2OH) (**Ir-3**). (**A**) Chemical structure of **Ir-3**. (**B**) PLIM image of hepatic lobules of an **Ir-3** administered mouse. CV: central vein, PV: portal vein, IR: intermediate region between CV and PV. Scale bar: 100 µm. (**C**) Phosphorescence lifetime of **Ir-3** and *p*O_2_ in hepatic lobules. **p* value < 0.05 by 2-tailed unpaired t-test. Error bar: S.D. The average *p*O_2_ were 16±3.2 mmHg in CV, 21±3.4 mmHg in IR, and 24±3.8 mmHg in PV. *N* = 4 ROI for CV, 8 for IR, and 7 for PV from two **Ir-3** administered mice. The $\tau_{p}^{0}$ and *k*_q_ of **Ir-3** in AML 12 cells were 4.80 µs and 5.16×10^3^ mmHg^-1^s^-1^.


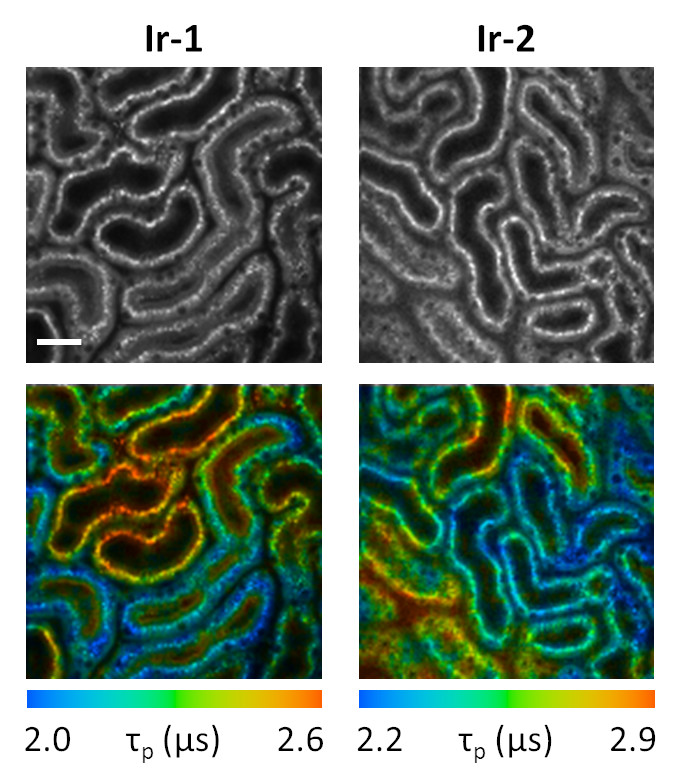


**Fig. S9** Intensity (top) and PLIM (bottom) images of renal surface of the **Ir-1** (left) and **Ir-2** (right) administered mouse. The average phosphorescence lifetimes in the renal tubules of **Ir-1** and **Ir-2** administered mice were 2.60±0.27 µs and 2.75±0.19 µs, respectively (the average of 16 points for four **Ir-1** administered mice and 5 points for two **Ir-2** administered mice**)**. These phosphorescence lifetimes were converted to the *p*O_2_ using the $k_{q}$ and $\tau_{p}^{0}$ values determined with HK-2 cells (Fig. S11) as 45±9.8 mmHg for **Ir-1** and 36±4.3 mmHg for **Ir-2**.


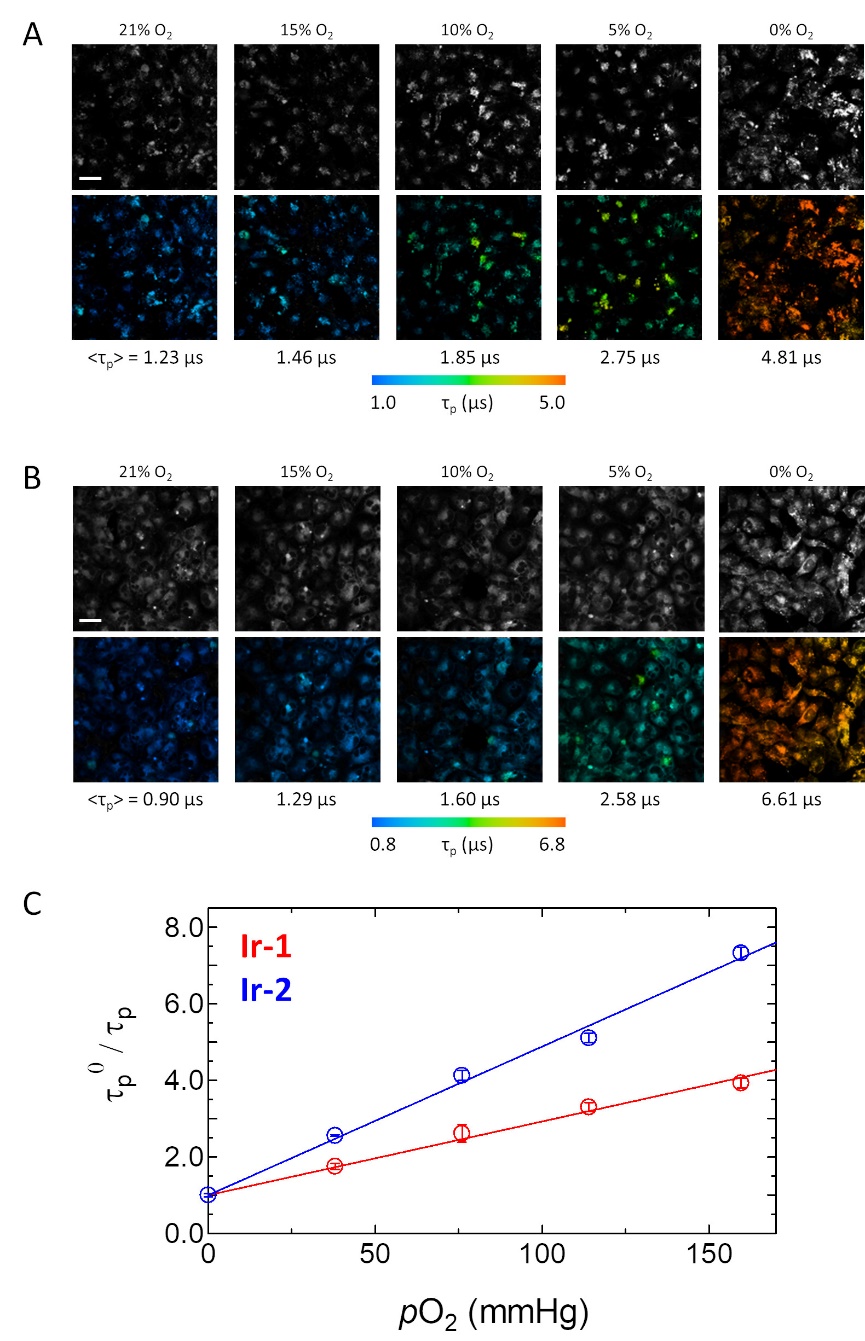


**Fig. S10** Calibration of phosphorescence lifetime using cultured HK-2 cells. (**A**, **B**) Phosphorescence intensity (upper figures) and PLIM (lower figures) images of HK-2 cells stained with **Ir-1** (**A**) and **Ir-2** (**B**) under different oxygen partial pressures in an incubator. The average phosphorescence lifetime is shown under each image. Cells are treated with Ant A to suppress cell respiration in 5-21% O_2_ experiments and Na_2_SO_3_ to remove dissolved oxygen in 0% O_2_ experiments. Scale bar: 50 µm. (**C**) Stern-Volmer plots of $\tau_{p}^{0}$/$\tau_{p}$ as a function of oxygen partial pressure for **Ir-1** (red) and **Ir-2** (blue) partitioned into HK-2 cells. Error bar: S.D. The $\tau_{p}^{0}$ and *k*_q_ in HK-2 cells were 4.81 µs and 4.00×10^3^ mmHg^-1^s^-1^ for **Ir-1** and 6.61 µs and 5.88×10^3^ mmHg^-1^s^-1^ for **Ir-2**.


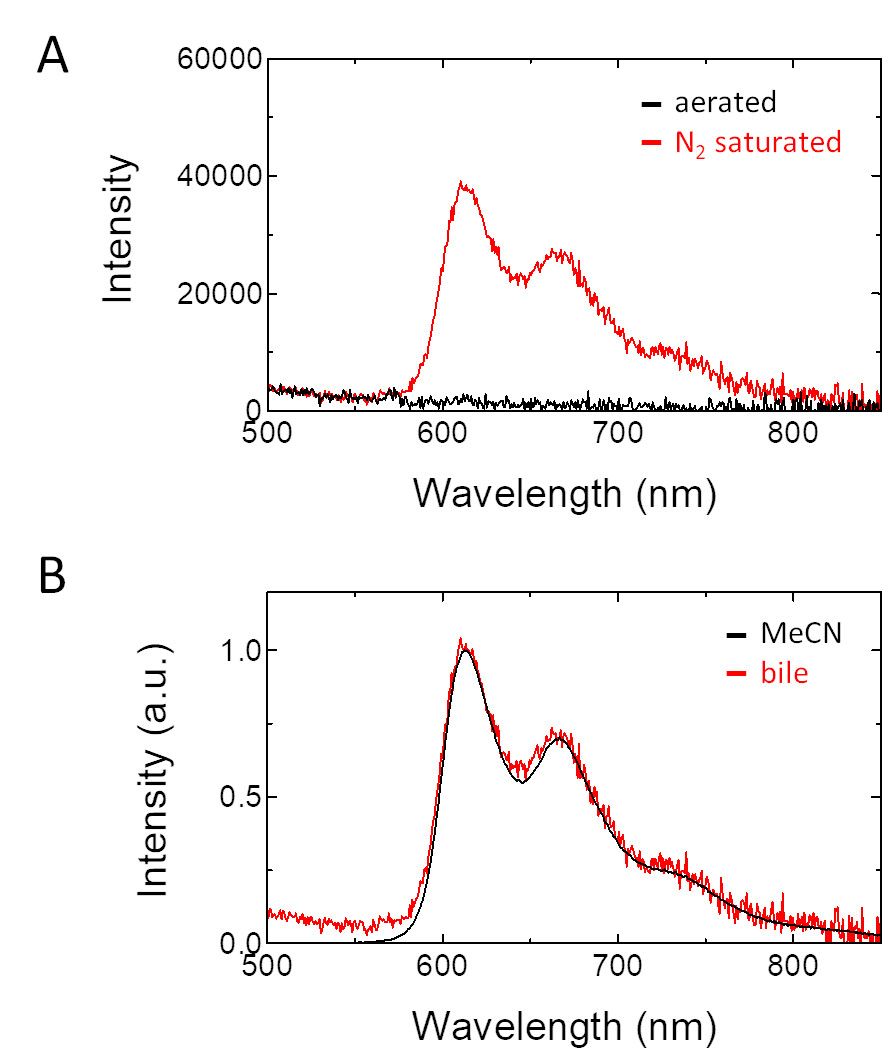


**Fig. S11** (**A**) Emission spectrum of the extract from bile 6 hours after **Ir-1** administration (red: in N_2_ saturated MeCN; black: in aerated MeCN). (**B**) Comparison of the emission spectrum (red) of the extract with the phosphorescence spectrum of **Ir-1** in MeCN (black). Normalized by the intensity of the emission maximum wavelength. Bile was collected 6 hours after the mouse was administered 250 nmol of **Ir-1**. The collected bile was separated with water and chloroform to extract an organic layer, and then dried under reduced pressure. The extract was dissolved in MeCN and the emission spectrum was measured.


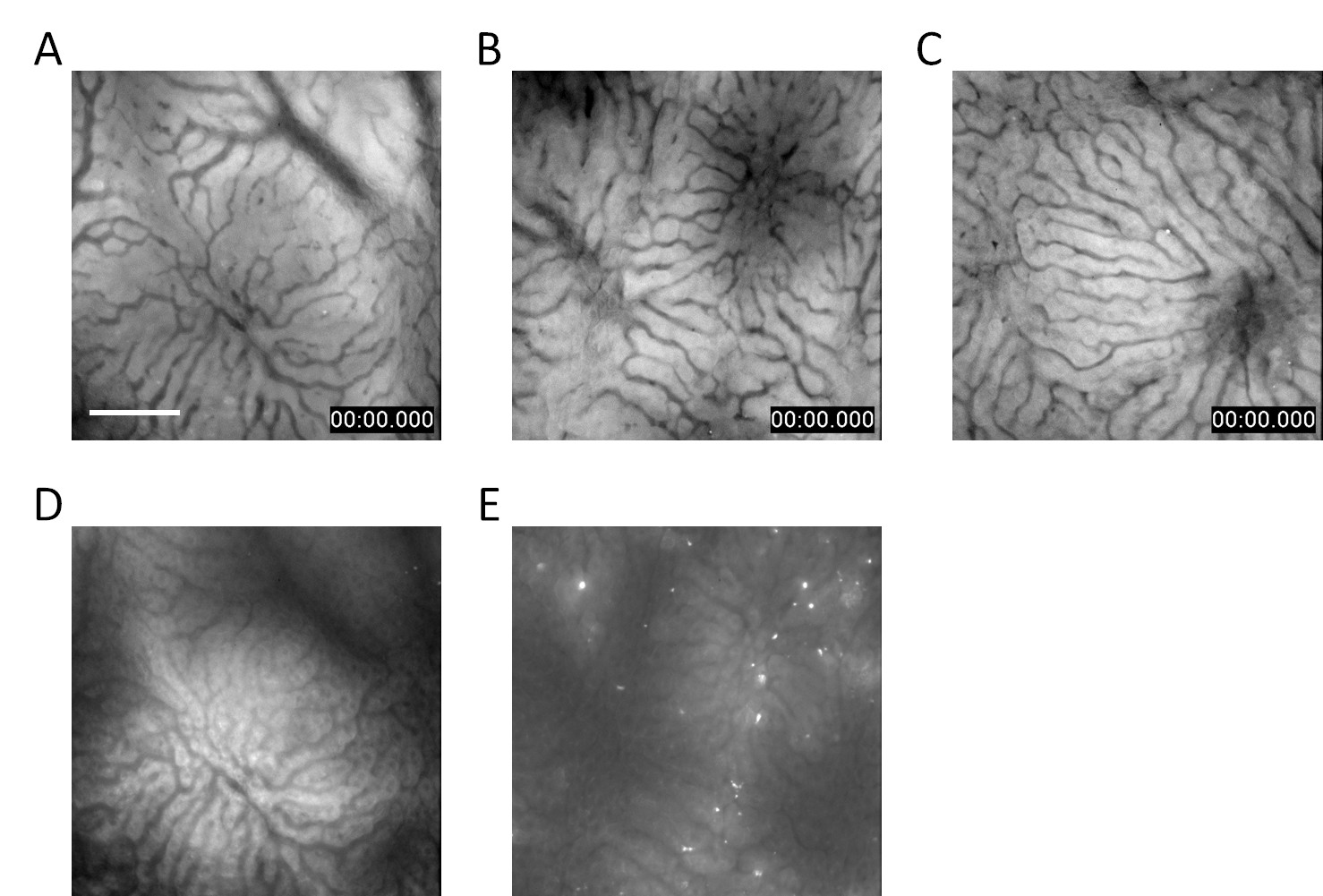


**Movie S1** (**A**, **B**, **C**) Emission video images of hepatic tissue around central vein of a mouse administered with **Ir-1** (**A**) and **Ir-2** (**B**), and a similar image without probe administration (**C**). *λ*_ex_: 450-500 nm, *λ*_em_: 515-565 nm, Scale bar: 100 µm. (**D**, **E**) Phosphorescence intensity images of the same area as image **A** (**D**) and **B** (**E**). *λ*_ex_: 400-440 nm, *λ*_em_: >590 nm.

**Liposome experiments.**  Lipid membranes of *L*-α-dimyristoyl-phosphatidylcholine (DMPC, Sigma-Aldrich) were generated as small unilamellar vesicles prepared by ethanol injection method.^1^ An ethanolic solution of DMPC (30 mM), and a DMSO stock solution of each probe (600 μM) were injected rapidly into Tris/HCl buffer or HEPES buffer. The final concentration of DMPC and the probe were 10 mM and 10 μM, respectively.

**Table S1** Phosphorescence lifetimes of **Ir-1** probe partitioned into DMPC

liposome under different **Ir-1** concentrations in Tris-HCl buffer.

**
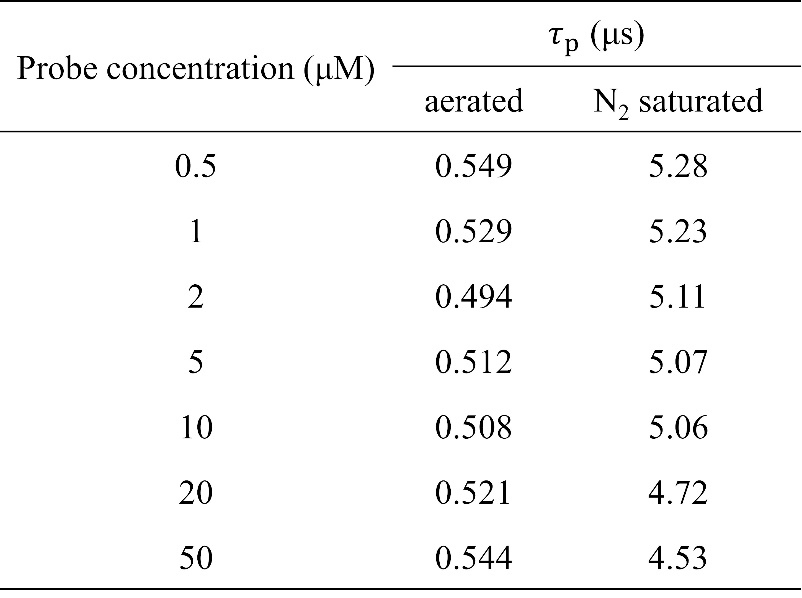
**

**Table S2** Phosphorescence lifetime of **Ir-1** and **Ir-2** partitioned into DMPC

liposome under different pH in Tris-HCl buffer or HEPES buffer at 37°C.

**
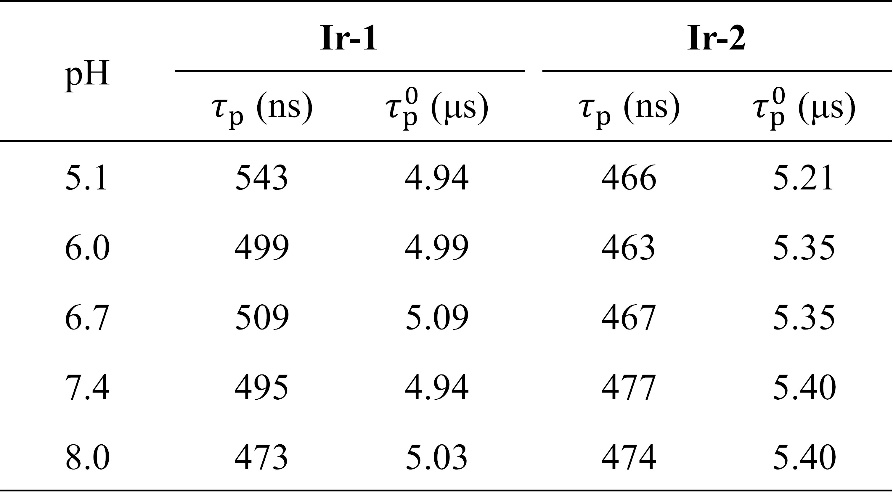
**

$\tau_{p}$ and $\tau_{p}^{0}$ denote the phosphorescence lifetime taken in aerated and N_2_ saturated solutions, respectively.

**Table S3** Phosphorescence lifetimes of **Ir-1** and **Ir-2** partitioned into DMPC

liposome with (1.0 mM) and without glutathione (GSH) in Tris-HCl buffer at 37°C.

**
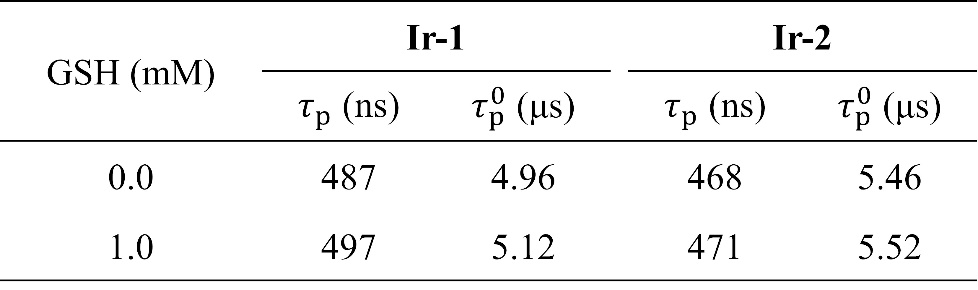
**

$\tau_{p}$ and $\tau_{p}^{0}$ denote the phosphorescence lifetime taken in aerated and N_2_ saturated solutions, respectively.

**Table S4** Representative molecular probes that have been used for O_2_ imaging of

cell spheroids.


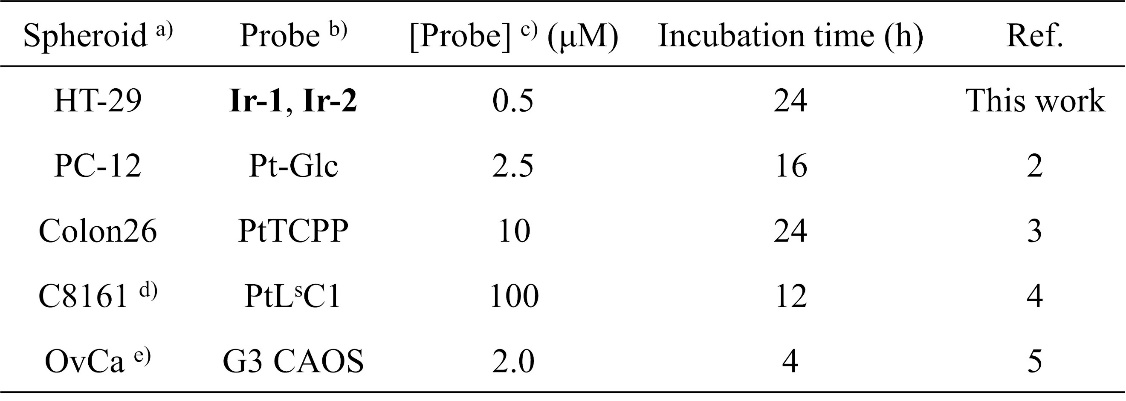


^a)^HT-29 (human colorectal adenocarcinoma cell), PC-12 (rat pheochromocytoma cell), Colon26 (mouse colon carcinoma cell), C8161 (human melanoma cell). OvCa (ovarian cancer cell). ^b)^Pt-Glc: glucose conjugate of Pt(II)-*meso*-tetrakis-(pentafluorophenyl)porphyrin (PtPFPP), PtTCPP: Pt(II)-5, 10, 15, 20-tetrakis-(4-carboxyphenyl)porphyrin, PtL^S^Cl: where L is a cyclometalating 3-di(2-pyridyl)benzene based ligand, G3 CAOS: click-assembled oxygen-sensing nanoconjugate with Pd(II) tetracarboxytetrabenzoporphyrin (PdTCTBP) as phosphorescent core. ^c)^probe concentration in the medium. ^d)^Spheroid size is larger than the other spheroids in this table. ^e)^Spheroid images are taken by using a single-photon-counting confocal microscope.

Materials and Methods

**Synthesis of BTPDM1 (Ir-1) and (btp-OH)_3_Ir (Ir-2)**


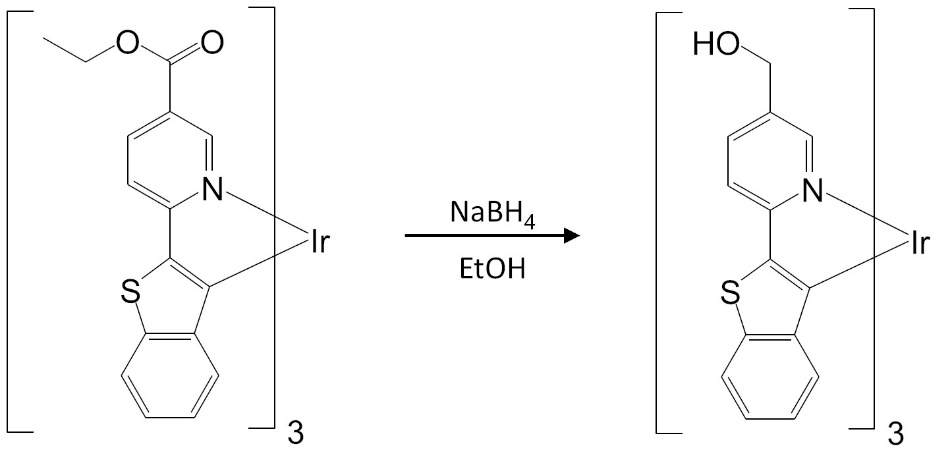


BTPDM1 (**Ir-1**) was synthesized as described previously.^6^ **Ir-2** was synthesized from BTP-COOEt which was prepared according to the procedure reported previously.^7^ To a solution of BTP-COOEt (100 mg, 0.10 mmol) in ethanol (20 mL) was added NaBH_4_ (200 mg, 5.3 mmol) quickly and then refluxed for 5 h. After cooling the solution was added to chloroform, then washed with distilled water. The organic layer was dried over sodium sulphate and evaporated to dryness under reduced pressure. Purification of crude product was performed by using a recycling preparative HPLC (LC-9225 NEXT, Japan Analytical Industry). The final product (**Ir-2**) was obtained as yellow powder (34 mg, 37 μmol, 39%). ^1^H NMR (400 MHz, DMSO-*d*_6_): δ 7.83.7.78 (m, 4H), 7.73-7.71 (m, 2H), 7.69-7.56 (m, 6H), 7.13-7.05 (m, 2H), 7.03-6.99 (m, 1H), 6.78-6.74 (m, 2H), 6.58 (t, *J* = 8.1 Hz, 1H), 6.35 (d, *J* = 7.8 Hz, 1H), 6.01 (dd, *J* = 16.9, 8.0 Hz, 2H), 5.19-5.10 (m, 3H), 4.26-4.22 (m, 2H), 4.20-4.10 (m, 4H). ESI-MS (positive): calcd. for C_42_H_30_IrN_3_O_3_S_3_ [M]^+^: 913.13, found: 913.2.

**Synthesis of (btp)_2_Ir(acac-2OH) (Ir-3)**


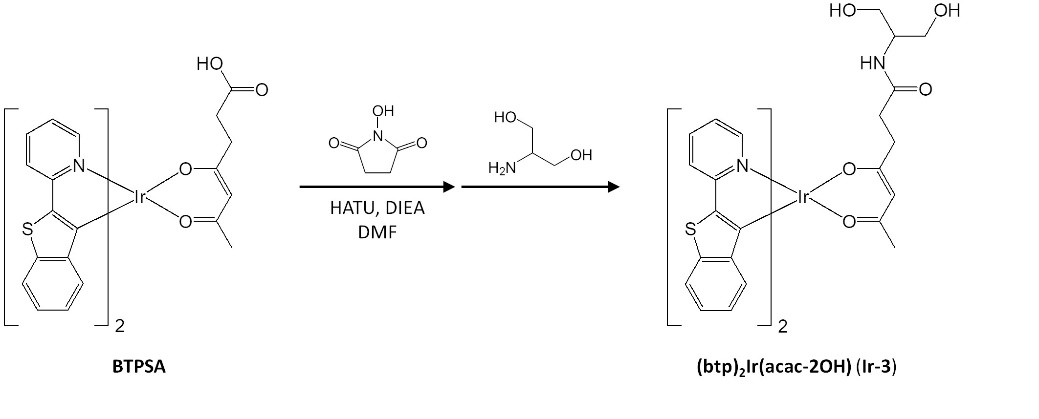


**Ir-3** was synthesized from BTPSA which was prepared according to the procedure reported previously.^6^ BTPSA (200 mg, 0.26 mmol), *N*-hydroxysuccinimide (70 mg, 0.61 mmol), *o*-(7-azabenzotriazol-1-yl)-*N,N,N’,N’*-tetramethyluronium hexafluorophosphate (HATU, 230 mg, 0.61 mmol), *N,N*-diisopropylethylamine (DIEA, 340 mL, 2 mmol) were dissolved in *N,N*-dimethylformamide (DMF, 3 mL). The solution was stirred for 5 h at room temperature under nitrogen, then 2-amino-1,3-propanediol (90 mg, 1 mmol) was added to the solution. The solution was stirred for 24 h at room temperature, and was evaporated to dryness under reduced pressure. The crude product was purified by aminopropyl-modified silica-gel column chromatography using chloroform/methanol (95:5, v/v) as eluent. The final product (**Ir-3**) was obtained as yellow powder (130 mg, 0.15 mol, 62%). ^1^H NMR (400 MHz, DMSO-*d*_6_): δ 8.39 (dd, *J* = 5.7, 23.1 Hz, 2H), 7.99-7.94 (m, 2H), 7.79-7.71 (m, 4H), 7.45 (d, *J* = 8.0 Hz, 1H), 7.28-7.23 (m, 2H), 7.06 (dd, *J* = 7.9, 7.2 Hz, 2H), 6.78 (dd, *J* = 8.0, 7.1 Hz, 2H), 6.05-6.01 (m, 2H), 5.33 (t, *J* = 5.7 Hz, 1H), 4.59-4.50 (m, 2H), 3.62-3.54 (m, 1H), 3.27-3.20 (m, 4H), 2.29-2.02 (m, 4H), 1.70 (s, 3H). ESI-MS (positive): calcd. for C_36_H_32_IrN_3_O_5_S_2_ [M]^+^: 843.14, found: 843.2.

**References**

1. Batzri, S., Korn, E. D. Single bilayer liposomes prepared without sonication, *Biochim. Biophys. Acta - Biomembr*. **298**, 1015–1019 (1973).
2. Dmitriev, R. I. *et al*. Small molecule phosphorescent probes for O_2_ imaging in 3D tissue models, *Biomater. Sci.* **2,** 853–866 (2014).
3. Kurokawa, H. *et al*. High resolution imaging of intracellular oxygen concentration by phosphorescence lifetime, *Sci. Rep.* **5**, 10657 (2015).
4. Raza, A. *et al*. Oxygen mapping of melanoma spheroids using small molecule platinum probe and phosphorescence lifetime imaging microscopy, *Sci. Rep*. **7**, 10743 (2017).
5. Nichols, A. J., Roussakis, E., Klein, O. J. & Evans, C. L. Click-assembled, oxygen-sensing nanoconjugates for depth-resolved, near-infrared imaging in a 3D cancer model, *Angew. Chem. Int. Ed*. **53**, 3671–3674 (2014).
6. Yoshihara, T. *et al*. Intracellular and in vivo oxygen sensing using phosphorescent Ir(III) complexes with a modified acetylacetonato ligand, *Anal. Chem.* **87**, 2710–2717 **(**2015).
7. Yoshihara, T., Hirakawa, Y., Nangaku, M. & Tobita, S. Hydrophilic Ir(III) Complexes for In vitro and In vivo Oxygen Imaging, *RSC Detection Science:* *Quenched-phosphorescence Detection of Molecular Oxygen: Applications in Life Sciences,* ed. Papkovsky, D. B. & Dmitriev, R. I. Royal Society of Chemistry, 2018, Chap. 4, pp.71-90.
